# Identification of Allosteric Inhibitors against Active Caspase-6

**DOI:** 10.1038/s41598-019-41930-7

**Published:** 2019-04-02

**Authors:** Agne Tubeleviciute-Aydin, Alexandre Beautrait, Jeffrey Lynham, Gyanesh Sharma, Alexei Gorelik, Ludovic J. Deny, Naoto Soya, Gergely L. Lukacs, Bhushan Nagar, Anne Marinier, Andrea C. LeBlanc

**Affiliations:** 1 Bloomfield Center for Research in Aging, Lady Davis Institute for Medical Research, Jewish General Hospital, 3755 Ch. Cote Ste-Catherine, Montreal, Quebec, H3T 1E2 Canada; 2 Department of Neurology and Neurosurgery, McGill University, 3775 University St., Montreal, Quebec, H3A 2B4 Canada; 3 Institute for Research in Immunology and Cancer, Université de Montréal, 2590, chemin de Polytechnique, Montreal, Quebec, H3T 1J4 Canada; 4 Department of Anatomy and Cell Biology, McGill University, 3640 University St., Montreal, Quebec, H3A 0C7 Canada; 5 Department of Biochemistry, McGill University, 3649 promenade Sir-William-Osler, Montreal, Quebec, H3G 0B1 Canada; 6 Department of Physiology and Biochemistry, McGill University, 3655 Promenade Sir-William-Osler, Montréal, Québec, H3G 1Y6 Canada

## Abstract

Caspase-6 is a cysteine protease that plays essential roles in programmed cell death, axonal degeneration, and development. The excess neuronal activity of Caspase-6 is associated with Alzheimer disease neuropathology and age-dependent cognitive impairment. Caspase-6 inhibition is a promising strategy to stop early stage neurodegenerative events, yet finding potent and selective Caspase-6 inhibitors has been a challenging task due to the overlapping structural and functional similarities between caspase family members. Here, we investigated how four rare non-synonymous missense single-nucleotide polymorphisms (SNPs), resulting in amino acid substitutions outside human Caspase-6 active site, affect enzyme structure and catalytic efficiency. Three investigated SNPs were found to align with a putative allosteric pocket with low sequence conservation among human caspases. Virtual screening of 57,700 compounds against the putative Caspase-6 allosteric pocket, followed by *in vitro* testing of the best virtual hits in recombinant human Caspase-6 activity assays identified novel allosteric Caspase-6 inhibitors with IC_50_ and *K*_i_ values ranging from ~2 to 13 µM. This report may pave the way towards the development and optimisation of novel small molecule allosteric Caspase-6 inhibitors and illustrates that functional characterisation of rare natural variants holds promise for the identification of allosteric sites on other therapeutic targets in drug discovery.

## Introduction

Caspases belong to a highly conserved family of cysteine proteases that have essential roles in programmed cell death, inflammation, differentiation, neuronal development and axon pruning^1^. Although apoptotic caspases are traditionally classified as initiators (Caspase-2, -8, -9 and -10) and effectors (Caspase-3, -6, and -7), data from Caspase-6 (Casp6) has challenged this classification. In addition to being activated by initiator and effector caspases, Casp6 can be activated by a unique intramolecular self-cleavage mechanism due to its longer intersubunit linker^2^. Moreover, the peptide substrate specificity of Casp6 is distinct from Caspase-3 (Casp3) and Caspase-7 (Casp7)^3–5^. Furthermore, inhibitors of apoptosis proteins that suppress Casp3 and Casp7 activity do not suppress Casp6 activity^6,7^. Finally, Casp3 or Casp7 activation in human embryonic kidney cells induces apoptosis whereas Casp6 activation does not^8,9^. These findings indicate that Casp6 displays unique structural features, substrate preference, and function compared to the other effector caspases.

Excess neuronal active Casp6 is associated with Alzheimer disease (AD) neuropathology and age-dependent cognitive impairment. Whereas active Casp6 is undetected in normal brains, it is highly abundant in post-mortem AD brains. Active Casp6 and Tau cleaved by Casp6 are present in neurofibrillary tangles (NFT), neuropil threads and neuritic plaques in the brain of mild, moderate, severe and very severe sporadic AD and in familial AD individuals^10–12^. Neuronal activation of Casp6 is associated with an increased production of amyloid beta peptide and caspase cleavage of Golgi-localised γ-ear-containing ARF binding protein 3 (GGA3), an inhibitor of the beta secretase^13–15^. In addition, Casp6 cleaves numerous substrates such as amyloid precursor protein^14,16–18^, tau^10^, α-tubulin^19^, valosin-containing protein^20^; a facilitator of misfolded ubiquitinated protein degradation, and actin-regulating postsynaptic density proteins spinophilin, α-actinin-1 and -4, and drebrin^19^, that may lead to neuronal dysfunction. Furthermore, Casp6 has been implicated in age-dependent cognitive impairment since levels of active Casp6 in the entorhinal cortex, the first region to be affected by AD^21^, inversely correlate with global cognitive, episodic, and semantic memory scores in individuals within normal cognitive range^22^. Knock-in mice that express a self-activated form of human Casp6 in the CA1 region of the hippocampus display age-dependent spatial and episodic memory impairment, in the absence of NFTs and amyloid plaques^23^. Furthermore, in nerve growth factor deprived mouse primary neuron cultures^24–26^, activation of Casp6 is associated with axonal degeneration, while Casp3 activation induces cell death. In wild type or mutant amyloid precursor protein-transfected human primary CNS neurons, Casp6 is activated in absence of Casp3 activation and leads to neuritic degeneration but not cell death^27^, consistent with observations in AD brains that Casp6 activity is present in neurons that do not have an apoptotic morphology^10^. Therefore, Casp6 represents a novel early therapeutic target in age-dependent cognitive impairment and AD.

Casp6 is a viable drug target. Casp6 null mice develop normally without any noticeable apoptotic defects^28^. Due to the role of Casp6 in B cell activation and plasma cell differentiation, these mice display increased antigen-stimulated production of antibodies; thus, Casp6 inhibition in AD patients may increase their immunity^29^. In humans, pro-Casp6 is not significantly expressed in foetal or adult brains^30^. Casp6 expression in peripheral tissues declines with age, but Casp6 expression remains relatively high in the gastrointestinal system^30^ raising concern that Casp6 inhibition may promote cancer. However, Casp6 involvement in tumour progression has been excluded in an inflammation-associated model of colon carcinogenesis^31^. Hence, Casp6 inhibition looks promising as a potential treatment for age-dependent cognitive impairment and AD.

Many competitive Casp6 inhibitors have been reported. Substrate mimetic peptide inhibitors were derived from the Casp6 cleavage site in transcription factor activator protein 2 isoform alpha^32^, lamin A^33^, Huntingtin (Htt)^34^, and synthetic caspase peptide substrates^35^. Most of these inhibitors contain an electrophilic warhead, aldehyde, fluoromethylketone or methyl vinyl sulfone^36^, which reacts with a catalytic cysteine. In addition, aza peptide epoxides^37^, Michael acceptors^38^, non-peptide inhibitors, sulfon amide isatin Michael acceptors^39^, 1,2,3-triazoles coupled to 2,3,5,6-tetrafluorophenoxy methyl ketone^40^, methylene blue and its derivatives^41^, have been shown to inhibit Casp6. Although most of these inhibitors display low nanomolar to micromolar potency against Casp6, some are toxic in mammals, have low blood brain barrier permeability, or lack selectivity. The catalytic site is highly conserved among the caspases, which challenges the development of highly selective Casp6 active site inhibitors.

A more promising approach is to target non-conserved allosteric sites^42^. Casp6 inhibition via zinc binding at exosite E (K36, E244, and H287)^43^, and phosphorylation of S257 at exosite D by ARK5 kinase^44–46^ represent two examples of natural Casp6 allosteric regulation. However, only exosite E has low conservation across the caspase family constituting an attractive allosteric site for Casp6 specific drug design. Despite the growing knowledge in the field of caspase allosteric regulation^42,47^, very few synthetic allosteric Casp6 inhibitors have been developed to date. A noncompetitive small peptide inhibitor identified through phage display screen against pro-Casp6 selectively inhibits Casp6 activity by trapping Casp6 in a tetrameric zymogen-like form^48^ but has poor cell penetration properties. Small molecule Casp6 inhibitors that bind at the dimer interface and stabilise the zymogen conformation^49^ do not bind to active Casp6. In addition, selective uncompetitive Casp6 inhibitors have been reported, but their action depends upon binding of an artificial peptide substrate limiting their therapeutic potential^50^. Active Casp6 inhibition is desirable because it accumulates before the onset of AD symptoms^11^. To our knowledge, no synthetic small molecule noncompetitive allosteric inhibitors of active Casp6 have been reported.

By investigating the activity of four rare natural Casp6 variants (Casp6-A34E, Casp6-E35K, Casp6-A109T, and Casp6-T182S) and employing X-ray crystallography along with molecular modelling, we have identified a putative allosteric pocket on mature active Casp6. An *in silico* screen of a diverse small molecules collection against this allosteric site identified two hits, S10G (IC_50_ = 4.2 µM) and C13 (IC_50_ = 13.2 µM) that inhibited Casp6 activity *in vitro* through a noncompetitive mechanism. Our data serves as a starting point for the development of a small molecule allosteric Casp6 inhibitors and also illustrates that screening of rare natural variants holds promise for the identification of allosteric sites on other therapeutic targets in drug discovery.

## Results

### Rare Casp6 variants display lower activity

**Figure 1:**
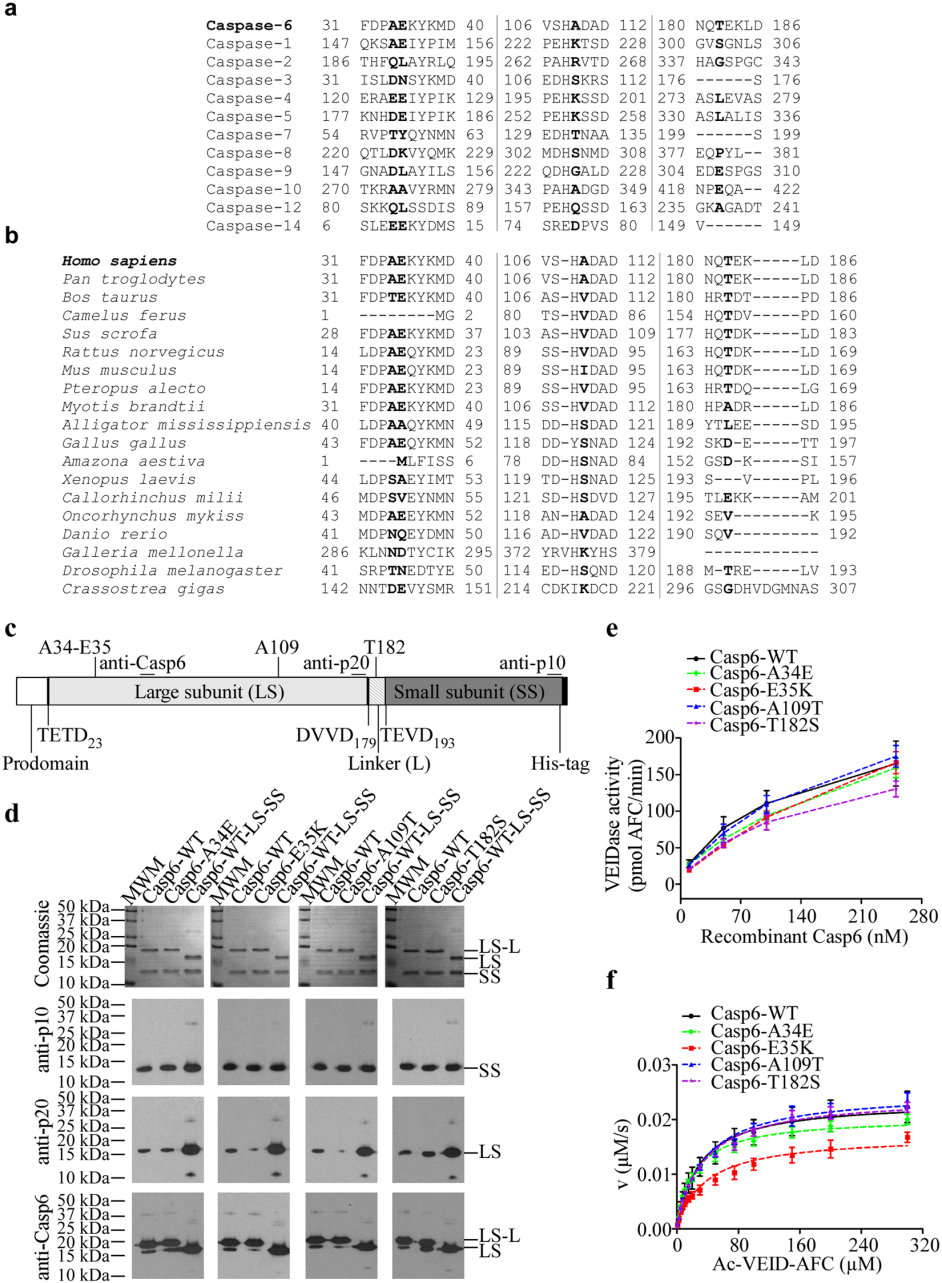
Activity of rare natural Casp6 variants. (**a**) Protein sequence alignment of 12 human caspases (**a**) and Casp6 from different species (**b**) showing A34, E35, A109, and T182 of human Casp6 and respective homologous residues of other caspases in bold. (**c**) A schematic representation of full-length recombinant pro-Casp6 and its domains, the position of Casp6 processing sites (TETD_23_, DVVD_179_, TEVD_193_), and the epitopes recognised by antibodies. Anti-p20 is a neoepitope antibody against cleaved Casp6 at D_179_ position. (**d**) Coomassie stain of SDS-PAGE (top panel) and western-blot analyses (three bottom panels) with Casp6 Pharmingen anti-p10, neoepitope 10630 anti-p20, and Santa Cruz anti-Casp6 (LS, LS-L) antibodies or antiserum of purified recombinant caspases showing large subunit with (LS-L) and without linker (LS), and small subunit (SS) of Casp6. Cropped gels and blots are shown here for clarity, full-length gels and blots are provided in Supplementary Information Fig. S12. (**e**) The activity of 10–250 nM active site titrated recombinant Casp6. (**f**) The relationship between initial reaction velocity (v) and substrate concentration of Casp6 catalysed Ac-VEID-AFC cleavage. Data were fitted to Michaelis-Menten equation using nonlinear regression and represent the mean ± standard deviation (SD) from at least three independent experiments.

In this study, we investigated how naturally occurring rare missense variants of human *CASP6* affect Casp6 activity and structure. Following our previous work, which described rare natural Casp6-R65W and Casp6-G66R variants with significantly impaired activity^51^, we examined four additional missense rare variants with single amino acid substitutions located remotely from Casp6 active site: Casp6-A34E, Casp6-E35K, Casp6-A109T, and Casp6-T182S (Table S1, Fig. S1). A34, E35, A109, and T182 are not conserved in 12 human caspases and in Casp6 from different species (Fig. 1a,b). Recombinant Casp6-WT and the four variants were expressed in *E. coli*, purified, and active site titrated (Fig. S2) for functional analyses. Like Casp6-WT, Casp6 variants, underwent complete self-processing at TETD_23_, and TEVD_193_ sites, and slight self-cleavage at the DVVD_179_ site, generating mainly large subunit with a linker (LS-L), small subunit (SS), and a minor amount of large subunit (LS) (Fig. 1c,d). Casp6 variants were catalytically active against Ac-VEID-AFC but Casp6-A34E, Casp6-E35K, and Casp6-T182S, displayed lower activity than Casp6-WT in enzyme concentration-dependent manner (Fig. 1e) whereas the activity of Casp6-A109T was comparable to Casp6-WT. Casp6-A34E, Casp6-A109T, and Casp6-T182S did not show significant changes in kinetic parameters, *K*_M_, and *k*_cat_, for Ac-VEID-AFC hydrolysis compared with Casp6-WT, whereas Casp6-E35K displayed slightly reduced *k*_cat_ and consequently showed ~2-fold reduced catalytic efficiency *k*_cat_/*K*_M_ (Fig. 1f and Table 1). These results suggest that E35K rare natural Casp6 substitution negatively affects Casp6 activity.

**Table 1:** Comparative catalytic efficiencies of Casp6 variants.

	*K*_M_ (µM)	*k*_cat_ (s^−1^)	*k*_cat_/*K*_M_ (s^−1^ M^−1^)
Casp6-WT	31.2 ± 6.7	2.27 ± 0.30	76,008 ± 18,626
Casp6-A34E	22.7 ± 0.8	2.04 ± 0.10	89,803 ± 6,453
Casp6-E35K	37.1 ± 4.9	1.71 ± 0.13	46,833 ± 7,641
Casp6-A109T	34.5 ± 1.8	2.51 ± 0.16	72,683 ± 2,529
Casp6-T182S	33.3 ± 1.2	2.42 ± 0.12	72,649 ± 1,192
Casp6-L200A	315.5 ± 162.0	0.05 ± 0.03	150 ± 24
Casp6-K284A	32.6 ± 10.8	2.0 ± 1.5	58,650 ± 30,269
Casp6-WT-LS-SS	24.8 ± 4.4	2.6 ± 0.4	109,148 ± 21,060
Casp6-L200A-LS-SS	196.2 ± 84.4	2.8 ± 0.8	15,155 ± 2,351
Casp6-K284A-LS-SS	27.7 ± 2.7	2.8 ± 0.2	99,882 ± 9,723

ND – not detectable, Data represent mean ± SD of at least three independent experiments.

**Figure 2:**
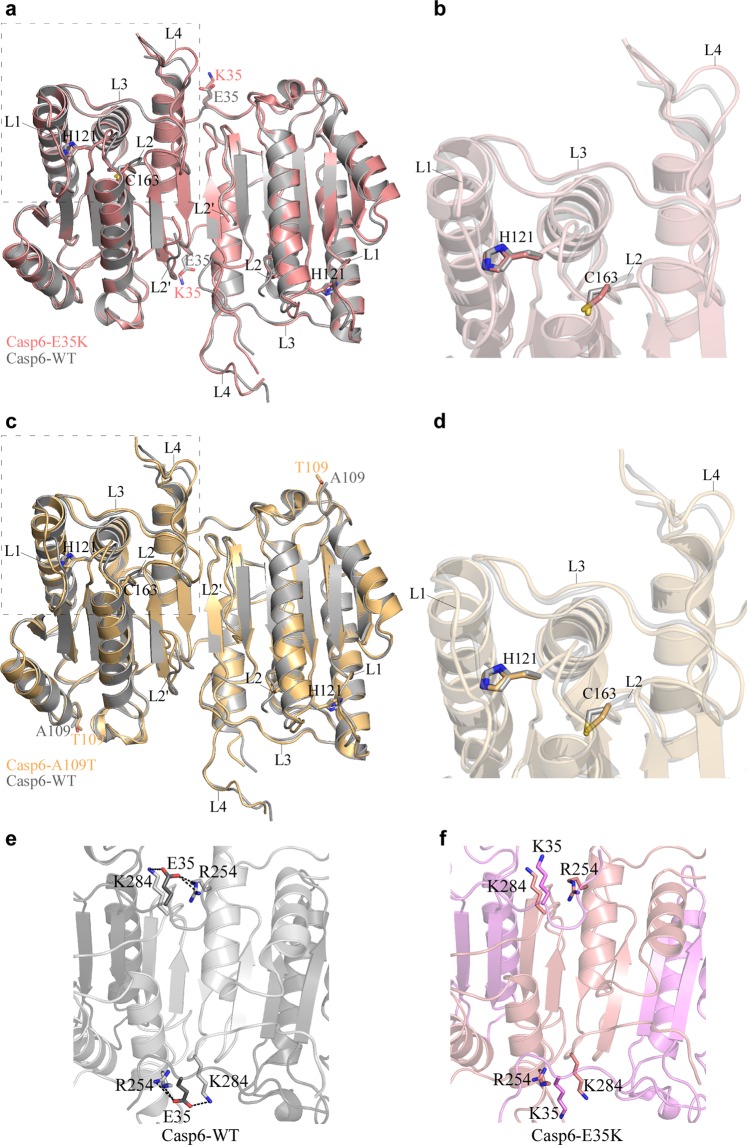
Structures of Casp6 rare variants. Crystal structures of mature apo Casp6-E35K (**a**,**b**, light pink) and Casp-A109T (**c**,**d**, light orange) superimposed on a mature apo structure of Casp6-WT (PDB: 2WDP, grey). The structures are represented as a cartoon with catalytic residues (H121, C163) and mutated residues (E35/K35, A109/T109) shown as sticks. L1, L2, L3, L4, and L2′ depict active site loops. Right panels (**b**,**d**) are the zoom-in images of boxed regions in left panels (**a**,**c**). The interaction of K35/E35 with R254 and K284 at the dimer interface of apo Casp6-WT crystal structure (**e**, dark grey LS, light grey SS) and crystal structure of the apo Casp6-E35K (**f**, light violet LS, light pink SS). Dotted black lines represent weak electrostatic E35 interaction (3.4–3.6 Å distance) with R254 and K284 in Casp6-WT.

To assess the effect of mutations on Casp6 structure and function, we solved apo mature crystal structures of Casp6-E35K and Casp6-A109T (Table S2) but crystallisation attempts were not successful for the two other Casp6 variants. Superposition of the Casp6-E35K or Casp6-A109T structure with a previously solved structure of the apo mature Casp6-WT (2WDP)^52^ did not reveal any significant changes in the active sites of these two variants (Fig. 2a–d), suggesting that the remote mutations, E35K or A109T, do not induce functional changes in the active site catalytic dyad C163-H121 or substrate binding loops of the mature unliganded Casp6. The Casp6-E35K crystal structure revealed some differences in the mutation site compared to the Casp6-WT (2WDP)^52^ structure. In Casp6-WT the E35 from the large subunit of one protomer forms a weak salt bridge with R254 from the small subunit of the other protomer (and vice versa), stabilising the dimeric Casp6 structure (Fig. 2e). By contrast, in the Casp6-E35K, the two salt bridges are replaced by two repulsive K35-R254 pairs (Fig. 2f). In addition, E35K substitution disrupts a salt bridge between E35 from the large subunit and K284 from the small subunit of the same Casp6-WT protomer (Fig. 2e,f), possibly further destabilising a functional oligomeric state of Casp6. The Casp6-A109T crystal structure did not reveal any significant changes around the mutation site, supporting our findings that A109T substitution does not have a significant impact on Casp6 activity.

### Identification of a putative allosteric pocket

**Figure 3:**
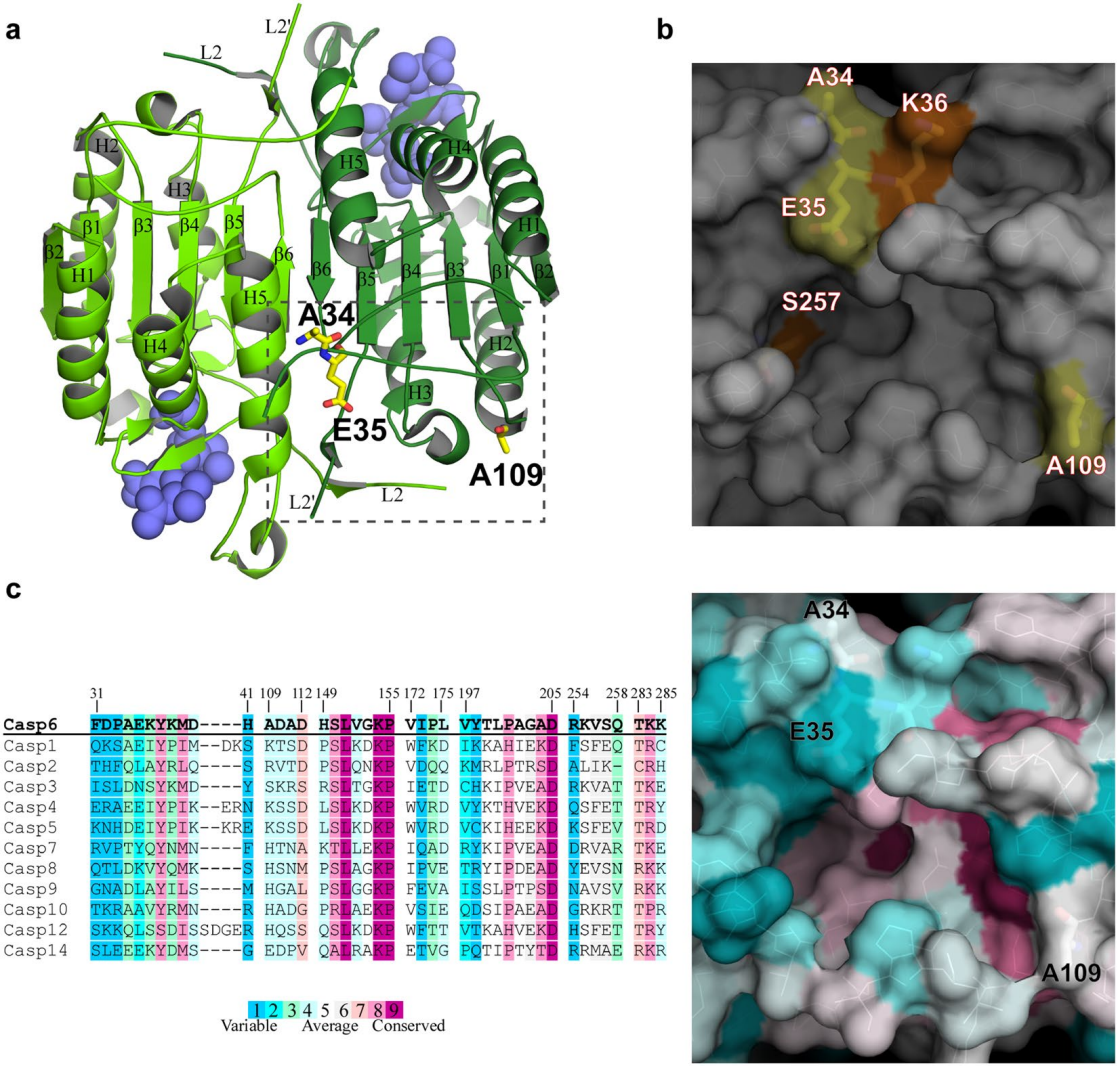
Putative Casp6 allosteric pocket. (**a**) SNP residues A34, E35, and A109 (yellow sticks) are highlighted in the putative allosteric pocket (boxed) from one protomer of the dimeric structure of the Ac-VEID-AFC-bound Casp6-WT (PDB: 3OD5). The protomers of dimeric Casp6 are shown in green and pale green, respectively, and the active-site bound Ac-VEID-AFC molecules are represented by blue spheres. Beta strands (β1–β6), alpha helices (H1–H5), and loops L2 and L2′ are labeled in both protomers. (**b**) Surface representation of the putative Casp6 allosteric pocket indicating positions of A34, E35, and A109 (yellow), and Casp6 allosteric regulation sites K36 and S257 (orange). (**c**) Amino acid sequence conservation of the putative allosteric pocket in the human caspase family (left panel) mapped on the 3OD5 crystal structure of Casp6 (right panel).

Visual inspection of four amino acid positions affected by the rare single nucleotide polymorphisms on the Ac-VEID-AFC-bound Casp6-WT structure (3OD5)^2^ revealed that the three amino-acids, A34, E35, and A109, located more than 30 Å away from the Casp6 catalytic site, line a well-defined pocket of low sequence conservation across the caspases (Fig. 3a–c). This pocket is made of residues from the N-terminal end, loops connecting β3 and β4 strands with helix 2 and helix 3, respectively, loop L2′ and C-terminal end of one protomer, and residues from L2 loop and helix 5 of the opposite protomer (Fig. 3a). Because of the size and favourable tertiary architecture of this pocket, we reasoned that it could represent a binding site capable of accommodating small organic compounds. To investigate this, we used an automated active site prediction tool that surveyed the entire surface of the Ac-VEID-AFC-bound Casp6-WT structure. This analysis validated this pocket as a potential druggable site and therefore supported our hypothesis that it could represent a putative allosteric site. Interestingly, the two residues S257 and K36, shown previously as Casp6 allosteric modulation points through phosphorylation and zinc binding^43–46^, are found inside and at the border of the putative allosteric pocket, respectively (Fig. 3b), further supporting the propensity of allosteric modulation in this region of Casp6.

### *In silico* screen for Casp6 allosteric inhibitors targeting the allosteric pocket

To identify candidate inhibitors of Casp6 activity, we performed a virtual screen by docking of small organic compounds targeting the putative allosteric site of the Ac-VEID-AFC-bound Casp6-WT structure. A total of 57,700 diverse molecules, originating from curated Chembridge and Sigma commercial libraries were screened. Subsequently to docking calculations, the poses of the best ranked molecules based on predicted binding energy were visually inspected to select the candidates that showed both favourable interactions within the putative allosteric site and ligand conformation of low energy. Finally, to ensure chemotype diversity a structural clustering was performed on the best candidates and a selection of 40 compounds was acquired (Tables S3 and S4).

### Verification of the inhibitory properties of *in silico* hits

To assess the potency of *in silico* hits, a fluorescence-based Ac-VEID-AFC cleavage assay was used to measure recombinant active Casp6 reaction rate in the absence or presence of 100 µM concentration of compounds. A 30% inhibition cut off was arbitrarily chosen for further investigation of putative inhibitors. Eleven of the forty compounds (27.5%) screened showed inhibition of Casp6 (Table 2, Fig. S3). AFC signal interference by compound intrinsic fluorescence was ruled out (Fig. S4). Casp6 inhibition was confirmed for seven compounds (C7, C13, C14, S2, S10, S11 and S13) using a colorimetric Ac-VEID-*p*NA cleavage assay (Table 2, Fig. S3). The four compounds (C16, C17, C25 and S6) that interfered with the *p*NA signal (Fig. S4) were tested through Casp6 cleavage of catalytically inactive Casp6-C163A. In the presence of 100 µM of C16, C17, and C25, the rate of Casp6-C163A prodomain cleavage by active Casp6 was reduced by ~30% compared to a DMSO control whereas 100 µM of S6 showed minimal inhibition of active Casp6 (Table 2, Fig. S3). The hits had IC_50_ values ranging from 13.2 µM to 289 µM (Table 2).

**Table 2:** Structure and inhibition data of hit compounds from Sigma (S) and Chembridge (C) libraries.

Compound	Structure	% Inhibition with 100 µM compound	IC_50_ (µM)^*^
C13		57.1 ± 4.7^a^ 86.7 ± 1.8^b^	13.2 ± 2.5
S10		59.7 ± 3.8^a^ 42.6 ± 3.4^b^	30.2 ± 6.8
C7		38.9 ± 5.5^a^ 92.0 ± 0.3^b^	44.1 ± 4.6
C16		33.3 ± 4.8^a^ 28.7 ± 7.4^c^	46.4 ± 3.3
S2		31.8 ± 4.8^a^ 52.9 ± 4.1^b^	90.0 ± 24.4
C25		34.9 ± 5.1^a^ 27.8 ± 14.0^c^	109.5 ± 15.4
S6		32.3 ± 4.3^a^ 6.3 ± 5.1^c^	123.0 ± 22.7
C14		34.0 ± 5.0^a^ 60.2 ± 6.5^b^	141.8 ± 14.8
S13		33.8 ± 12.1^a^ 41.9 ± 8.2^b^	159.0 ± 27.7
C17		30.3 ± 1.7^a^ 23.8 ± 20.0^c^	233.4 ± 28.1
S11		30.0 ± 6.0^a^ 56.3 ± 2.3^b^	289.0 ± 60.9

^a^Fluorescence assay, ^b^colorimetric assay, ^c^Casp6-C163A cleavage assay, ^*^IC_50_ values were determined using fluorescence assay.

To probe a preliminary structure-activity relationship (SAR) of the two most active hits, S10 and C13, a few commercially available analogues (Table S5) were tested using the fluorescence-based activity assay. Twelve of the eighteen S10 analogues tested at 100 µM (S10B-S10I, S10K-S10M, and S10P) showed ≥50% inhibition of Casp6 (Table 3, Fig. S3). Compounds S10B–S10F, S10H, S10M, and S10P were verified as Casp6 inhibitors using the colorimetric assay, and compounds S10G, S10I, S10K, and S10L were verified using the Casp6-C163A cleavage assay (Table 3, Fig. S3). These hits had IC_50_ values ranging from 4.2 μM to 79.9 μM (Table 3). Compounds S10G, S10K, and S10L had IC_50_ values ~3.8-fold lower than the IC_50_ of S10 (30.2 μM). Interestingly, compound S10P, which is devoid of the critical pyrimidinetrione scaffold known as being a PAINS^53^, retained some activity with an IC_50_ of 80 µM.

**Table 3:** Structure and inhibition data of S10 and C13 analogues.

Compound	Structure	% Inhibition with 100 µM compound	IC_50_ (µM)^*^
S10G		98.6 ± 2.4^a^ 99.1 ± 1.5^c^	4.2 ± 0.3
S10L		89.0 ± 3.0^a^ 90.5 ± 5.2^c^	4.4 ± 0.4
S10K		81.7 ± 2.0^a^ 81.7 ± 3.4^c^	7.6 ± 0.8
S10D		96.3 ± 1.6^a^ 53.3 ± 7.8^b^	9.8 ± 1.7
S10B		92.0 ± 6.7^a^ 64.4 ± 14.7^b^	10.5 ± 2.4
S10C		96.8 ± 0.7^a^ 62.9 ± 6.8^b^	11.0 ± 1.3
S10E		94.6 ± 1.1^a^ 46.8 ± 7.4^b^	12.8 ± 1.8
S10F		93.7 ± 0.8^a^ 52.5 ± 6.5^b^	12.9 ± 1.3
S10M		81.3 ± 3.3^a^ 43.7 ± 3.1^b^	16.4 ± 3.1
S10H		92.3 ± 2.7^a^ 53.4 ± 10.4^b^	16.5 ± 2.3
S10I		74.3 ± 5.8^a^ 81.8 ± 4.6^c^	16.8 ± 2.2
S10P		59.3 ± 2.5^a^ 44.7 ± 4.7^b^	79.9 ± 9.9
C13G		55.5 ± 8.8^a^	21.5 ± 2.0
C13F		43.2 ± 10.9^a^	27.0 ± 1.6
C13C		24.7 ± 1.7^a^	719 ± 76^**^
C13A		21.8 ± 2.5^a^	1545 ± 261^**^
C13B		2.3 ± 3.1^a^	1922 ± 300^**^
C13D		5.5 ± 4.1^a^	2047 ± 57^**^
C13E		−8.3 ± 1.7^a^	3370 ± 678^**^

^a^Fluorescence assay, ^b^colorimetric assay, ^c^Casp6-C163A cleavage assay, ^*^IC_50_ values were determined using fluorescence assay, ^**^approximate IC_50_ values calculated from not-perfectly fitted dose-response curves due to limited compound solubility (C13A, C13C) or very low compound potency at the highest concentration tested (C13B, C13D, C13E).

In the C13 series, seven analogues were tested, most of them exploring various replacements of the di-hydrobenzofuran right hand side-chain. While two of them (C13F and C13G) retained the activity seen with C13, most of them were found significantly less potent (Table 3).

### Compound S10G and C13 inhibit Casp6 through a noncompetitive mechanism

**Figure 4:**
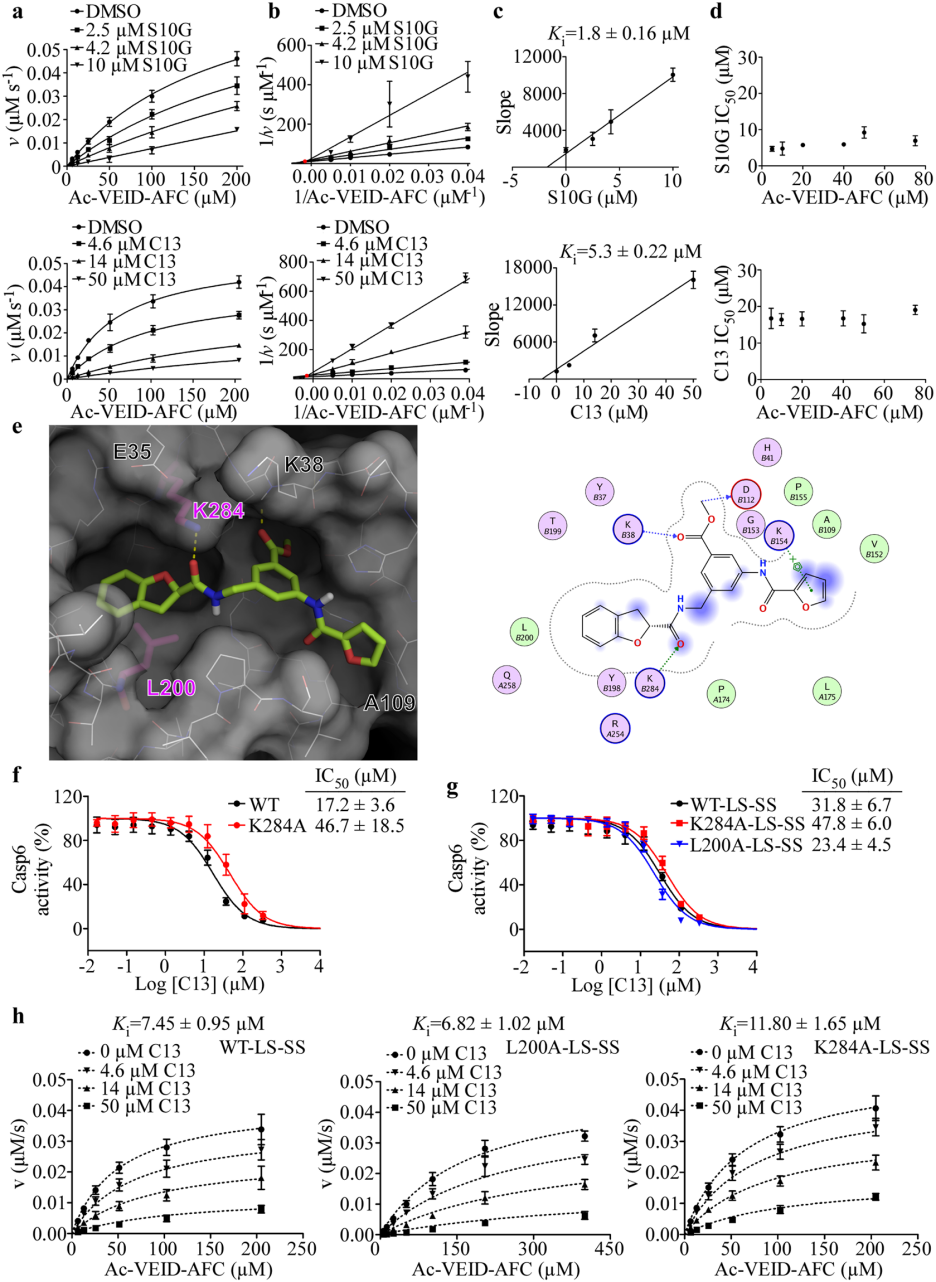
Compounds S10G and C13 inhibit Casp6 through a noncompetitive mode of inhibition. (**a**) Michaelis-Menten kinetic plots of Casp6 enzymatic activity in presence of 1% DMSO or increasing doses S10G (top) and C13 (bottom). Data points represent mean ± SD of three independent experiments. (**b**) Lineweaver-Burk plots of Casp6 inhibition with S10G (top) and C13 (bottom). Red circle indicates the point where lines converge. (**c**) The relationship between the slopes of lines in Lineweaver-Burk plot analyses in panel (b) and inhibitor concentration. (**d**) Plots of IC_50_ values for S10G (top) and C13 (bottom) with varying concentrations of Ac-VEID-AFC substrate. Data points represent mean ± SD from three independent experiments for each substrate concentration. (**e**) Binding model of C13 into CASP6 putative allosteric site, derived from molecular docking. Left: C13 (green sticks) occupies the putative allosteric site (white surface and lines; Ala-mutated residues L200 and K284 shown as purple sticks). Right: predicted interactions with Casp6 allosteric pocket residues (pink balls polar, circled blue or red for basic and acidic, respectively. Green balls = hydrophobic. Dotted-arrow: blue and green for backbone and sidechain hydrogen-bond interactions, respectively. Blue shade = solvent exposed area). Dose-response curves of C13 for Casp6-WT and Casp6-K284A (**f**), and copurified LS and SS of Casp6-WT-LS-SS, Casp6-L200A-LS-SS, and Casp6-K284A-LS-SS (**g**). Data represent mean ± SD from at least three independent experiments. (**h**) Global fit of kinetics data to a noncompetitive (mixed) inhibition model for Casp6-WT-LS-SS (left panel, R^2^ = 0.9635), Casp6-L200A-LS-SS (middle panel, R^2^ = 0.9699), and Casp6-K284A-LS-SS (right panel, R^2^ = 0.9794) in the presence of 0–50 µM C13. Data points on the graph represent the mean ± SD, and calculated *K*_i_ values represent mean ± standard error (SE) from three independent experiments.

The inhibition of Casp6 activity with S10G and C13 was reversible (Fig. S5). To determine mode of inhibition, kinetic analyses were conducted on Casp6 using S10G and C13 (Fig. 4a). In the Lineweaver-Burk plot, lines of best fit converged at a single point above the x-axis and left from the y-axis, which is characteristic of noncompetitive inhibition^54^ (Fig. 4b). The dissociation constant (*K*_i_) of S10G was 1.8 µM, which was approximately 3-fold lower than C13 (*K*_i_ = 5.3 µM) (Fig. 4c). Moreover, IC_50_ values determined using six different Ac-VEID-AFC concentrations did not show significant differences, excluding competitive inhibition mode (Fig. 4d).

### Binding mode analysis of C13

The predicted C13 binding pose, derived from molecular docking, and the two-dimensional ligand interaction diagram (Fig. 4e) revealed several potentially important interactions between the C13 and the Casp6 putative allosteric pocket residues. The K38 backbone amide formed a hydrogen bond with the carbonyl of C13 methyl ester group. Furthermore, the model proposed that the terminal charged amine of K154 sidechain may be involved in a π-cation interaction with the C13 furan moiety. The model also revealed a pretty tight pocket surrounding the furan ring, consistent with the 117-fold reduction in the potency of the benzyl analogue C13A compared to C13 (Fig. S6, Table 3). In addition, the K284 sidechain formed a hydrogen bond with the carbonyl group of the methylamide linker of C13, and the L200 engaged in a hydrophobic interaction with the C13 dihydrobenzofuran end (Fig. 4e). Analogues either lacking the carbonyldihydrobenzofuran moiety (C13B) or wherein the dihydrobenzofuran group has been replaced by a *tert*-butyl group (C13E) were too short to optimally fill all subpockets of the allosteric site occupied by C13 and showed low potency against Casp6 (Table 3). Replacement of the dihydrobenzofuran moiety in C13 with a 2,3-dimethyl-1H-indole group in C13C and 5-methyl-3-phenyl-isoxazolyl group in C13D resulted in a non-optimal docking poses and a 55-fold and a 155-fold increase in IC_50_ for Casp6-WT, respectively, compared to C13 (Table 3). On the other hand, the replacement of dihydrobenzofuran group in C13 with a 4-chlorophenyl-cyclobutyl group in C13F, and 3,5-dimethylbenzoyl group in C13G resulted in a rather moderate 2-fold increase in IC_50_ for Casp6-WT, consistent with the reasonable docking poses obtained with this pair of compounds in the Casp6 allosteric pocket (Fig. S6).

To determine the importance of C13 interactions with L200 and K284 for C13 binding in the Casp6 allosteric pocket, we have generated Casp6-L200A and Casp6-K284A mutants and investigated inhibition with C13. In contrast to Casp6-WT, purified Casp6-L200A and Casp6-K284A underwent poor self-processing when overexpressed in *E. coli* from a construct encoding a full-length Casp6 (Fig. S5). Casp6-L200A partially self-processed at the TETD_23_ site generating full-length Casp6 and Casp6 without a prodomain (ΔCasp6). Casp6-K284A underwent complete processing at the TETD_23_ site but, in contrast to Casp6-WT, it only partially self-cleaved at the TEVD_193_ site generating ΔCasp6, LS-L and SS fragments (Fig. S5). Consistent with the incomplete self-processing, active site titration of Casp6-L200A did not yield any activity, whereas Casp6-K284A had only ~20% of active sites, in contrast to nearly 100% active sites determined for Casp6-WT samples (Fig. S2). Fully processed forms of Casp6-WT-LS-SS, Casp6-L200A-LS-SS, and Casp6-K284A-LS-SS were obtained by co-expression and purification of LS and SS subunits of WT, L200A or K284A Casp6 from *E. coli* (Fig. S5). Active site titration with zVAD-FMK inhibitor indicated that both Casp6-K284A-LS-SS and Casp6-WT-LS-SS had more than 80% active sites, and Casp6-L200A-LS-SS had ~178% active sites (Fig. S2), suggesting that zVAD-FMK may not be potent enough to react with Casp6-L200A-LS-SS active sites effectively. The kinetic parameters, *K*_M_ and *k*_cat_, for Ac-VEID-AFC hydrolysis were similar between Casp6-WT and Casp6-K284A as well as Casp6-WT-LS-SS and Casp6-K284A-LS-SS suggesting that K284A mutation does not affect Casp6 catalytic function (Table 1). In contrast, Casp6-L200A and Casp6-L200A-LS-SS displayed 10- and 8-fold higher *K*_M_ values compared to Casp6-WT and Casp6-WT-LS-SS, respectively. Furthermore, the catalytic function of unprocessed Casp6-L200A was almost completely diminished as reflected by a 45-fold reduction in *k*_cat_ compared to Casp6-WT. In contrast, fully processed Casp6-L200A-LS-SS had a turnover number comparable to that of Casp6-WT-LS-SS (Table 1). Taken together these results suggest that K284A and L200A mutations negatively affect Casp6 self-processing and L200A impairs peptide substrate binding.

The C13 compound displayed ~2.7-fold higher IC_50_ value for Casp6-K284A mutant than Casp6-WT (Fig. 4f). Compared to Casp6-WT-LS-SS, the IC_50_ of C13 was 1.5-fold higher and 1.4-fold lower for the fully processed Casp6-K284A-LS-SS and Casp6-L200A-LS-SS, respectively (Fig. 4g). In the presence of increasing concentrations of C13 compound, an increase in apparent *K*_M_ and a decrease in apparent V_max_ of Ac-VEID-AFC hydrolysis was observed for Casp6-WT-LS-SS, Casp6-L200A-LS-SS, and Casp6-K284A-LS-SS, which indicates a noncompetitive (mixed) model inhibition (Fig. S5). Global fitting of Ac-VEID-AFC hydrolysis velocity data at increasing substrate and C13 concentrations to the velocity equations for different inhibition modalities revealed the best fit for a noncompetitive (mixed) model inhibition for Casp6-WT-LS-SS, Casp6-L200A-LS-SS, and Casp6-K284A-LS-SS (Fig. 4h, Fig. S7). The calculated *K*_i_ value of C13 for Casp6-L200A-LS-SS (6.8 µM) was not significantly different from the *K*_i_ for Casp6-WT-LS-SS (7.5 µM), whereas *K*_i_ for Casp6-K284A-LS-SS (11.8 µM) was 1.6-fold higher compared to the *K*_i_ for Casp6-WT-LS-SS (Fig. 4h) suggesting that K284 may play a minor role in binding C13 in the Casp6 allosteric pocket.

For validation purposes, we have synthesised C13 and C13G in our laboratory (Fig. S8). Compared to C13 and C13G obtained from commercial sources, our synthesised compounds displayed lower potency for Casp6.

### Evaluation of S10G binding to Casp6 using Hydrogen-Deuterium Exchange Mass Spectrometry (H/DX-MS)

**Figure 5:**
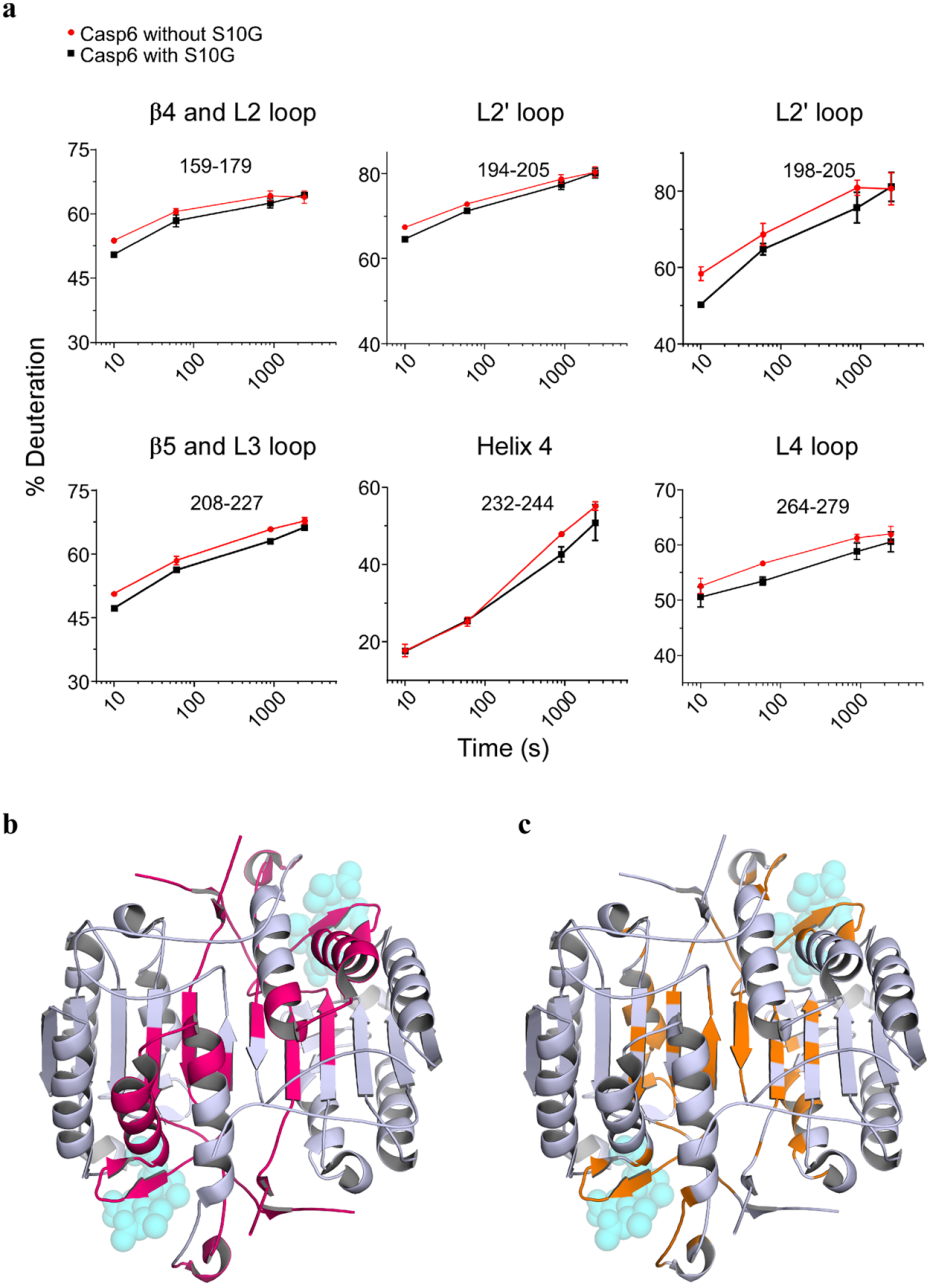
S10G and Casp6 interaction sites. (**a**) Representative deuterium uptake plots for peptic peptides of Casp6 incubated without S10G (red) and with S10G (black). Data points represent mean ± SD from three independent experiments. (**b**) Representative peptic peptides from panel (a) are mapped (pink area) on the CASP6 crystal structure (PDB: 3OD5) and compared to potential binding sites of S10G, as predicted by molecular docking (**c**, orange area). For indication purposes, Ac-VEID-AFC substrate, as found in CASP6-bound structure, is shown as translucent spheres. Same point of view as in Fig. 3.

In light of the inconclusive results from site-directed mutagenesis impact on C13 inhibition, a biophysical approach was undertaken using S10G. To evaluate the changes in the Casp6 conformational dynamics affected by S10G binding, we have incubated fully maturated Casp6 with and without S10G in deuterium buffer for between 10 s and 40 min to allow H/D exchange, followed by pepsin digestion and MS for peptide mass analysis. A heat map representing relative differential deuteration profiles of various structural elements in Casp6 over the 40 min H/D exchange time was obtained for Casp6 incubated with and without S10G (Fig. S9). Overall, more than 60% of the backbone amide hydrogens underwent less than 30% H/D exchange within 10 s, indicating that Casp6 is a well-folded and dynamically stable protein. Lower H/D exchange profile was observed for the buried regions of the Casp6 core structure, whereas L2 and L2′ loops displayed highest H/D exchange levels suggesting that these regions are more flexible and solvent exposed in the fully mature Casp6. The peptic peptide coverage was nearly 100% for both Casp6 incubated with and without S10G (Fig. S9), allowing accurate detection of differences in conformational flexibility of Casp6 induced by S10G. Incubation of Casp6 with a 5-fold molar excess of S10G, resulted in a statistically significant decrease in H/D exchange in several peptic peptides (Fig. 5a, Fig. S10), which were mapped on the three-dimensional structure of the active-site liganded Casp6 (Fig. 5b), and cover regions in L2, L2′, L3 and L4 loops, β4 and β5 strands, helix 4, and loop connecting to the helix 5 N-terminal end. Overall, observed changes in deuterium uptake in the presence and absence of S10G for these regions were less than 10%, with the biggest differences detected for the L2′ and L4 loops. *In silico* docking of S10G onto the Casp6 structure indicated several putative S10G binding sites (Fig. 5c), which partially overlapped with H/DX-MS results, suggesting that S10G may interact with the substrate binding loops and the dimer interface.

## Discussion

Aiming to identify novel allosteric sites in Casp6, we have investigated how human Casp6 rare non-synonymous missense SNPs resulting in substitutions away from the Casp6 active site, affect Casp6 catalytic efficiency and structure. In contrast to our previous study describing significant loss of activity in Casp6-R65W and Casp6-G66R rare SNP variants^51^, in the four A34E, E35K, A109T, and T182S remote SNPs investigated here, only the E35K slightly decreased Casp6 catalytic efficiency for a peptide substrate. The crystal structure of apo Casp6-E35K did not reveal hints that could have elucidated a potential mechanism responsible for reduced Casp6 activity. Although E35K substitution may lead to the disruption of few intersubunit salt bridges within Casp6 dimer, we did not observe decreased *in vitro* stability of Casp6-E35K compared to Casp6-WT or dissociation of enzyme subunits during protein purification (Fig. S11) recently described for Caspase-9 mutant^55^, which could provide an explanation for the enzyme inactivation.

Located more than 30 Å away from either active site of the Casp6 dimer, the N-terminal loop E35 is found in the neighbourhood of K36, which together with E244 and H287 forms Zn^2+^-binding exosite^43^ next to the postulated allosteric pocket. Casp6 inhibition with Zn^2+^ binding at this exosite, as well as findings that natural substitution E35K and alanine mutants of Zn^2+^-coordinating residues^43^ significantly reduce Casp6 catalytic efficiency suggest that this area, remote from Casp6 active sites, is important for Casp6 activity. Additionally, S257, the single reported Casp6 phosphorylation site, whose phosphorylation by ARK5 kinase allosterically inhibits Casp6^44–46^, is located at the postulated Casp6 allosteric pocket, which further supports our hypothesis that small molecules binding in the pocket surrounded by A34E, E35K, and A109T SNPs, could result in inhibition of Casp6 activity. A recent study investigating Casp3 phosphorylation sites, S150 and T152 residues in the C-terminal loop of helix-3 (H3CL), a conserved region also found in the putative allosteric pocket of Casp6, revealed a network of interactions, which couple changes in the allosteric H3CL site to both active sites of the Casp3 dimer^56^. The localised changes in the H3CL region were shown to propagate through the helix-3 to the connected β-strand containing catalytic histidine as well as affect the active site loop bundle of the opposite protomer^56^. The authors of the latter study made an excellent insight that the H3CL region in Casp6 is connected to the helix-3, part of which undergoes conformational change into β-strands when Casp6 transits from inactive extended helical to active canonical conformation^57,58^. This suggests that the putative Casp6 allosteric pocket may indeed have a more central role in regulating Casp6 activity, and perhaps an important biological ligand for this allosteric site remains to be uncovered since Casp6 is not known to be phosphorylated at the S150 site identical to the one in Casp3.

The important structural components of the putative Casp6 allosteric pocket are L2 and L2′ loops. Upon cleavage of the intersubunit linker, the released C-terminal region of the large subunit, and the N-terminal region of the small subunit, become L2, which contains catalytic C163, and L2′ loops, respectively. In a fully mature, active-site liganded conformation of Casp6, a region of the L2 loop of one protomer interacts in the putative allosteric pocket with a segment of the L2′ loop of the opposite protomer, which could represent a putative interprotomer coupling between the postulated allosteric pocket and the active site in the Casp6 dimer. Casp6 allosteric inhibition is often achieved through the conformational changes in the L2′ and L2 loops. Phosphorylation of Casp6 at the S257 results in a steric clash with L2′ loop, which folds over the dimer interface as seen in the apo mature form of Casp6, resulting in misalignment of the substrate binding loops^44,45^. Small molecule inhibitors bind procaspase-6 allosteric site at the dimer interface and interact with residues in the L2 loop stabilising Casp6 zymogen-like conformation^49^. Likewise, small molecule covalent inhibitors bind at the analogous allosteric site at the dimer interface of Casp1^59^, 5^60^, 3 and 7^61^, and lock L2′ loop in inactive zymogen-like conformation in Casp7, preventing substrate binding^61^. Our data indicate that Casp6 L2′ loop L200A mutant almost completely abolishes Casp6 self-processing and catalytic efficiency, consistent with the previous report for the analogous I213A mutation in the L2′ loop of Casp7^62^, which revealed an essential role of L2′ loop in providing a platform for the proper arrangement of the substrate-binding groove in caspases. Interestingly, this was the first study^62^ to propose that small-molecule binding to the area occupied by I213 of L2′ loop in Casp7, which overlaps with the putative allosteric pocket of Casp6, could provide a novel strategy for modulating caspase activity.

Virtual screening of small molecules against active human Casp6 putative allosteric pocket followed by *in vitro* evaluation of Casp6 inhibition identified two Casp6 hit inhibitors, S10G and C13. Both compounds display low micromolar *K*_i_ and a noncompetitive mode of Casp6 inhibition, suggesting that they bind to an allosteric rather than a catalytic site, which validates our approach. Although our study lacks experimental evidence supporting the direct binding of C13 and S10G to the postulated allosteric pocket, the preliminary SAR of C13, where the core of the C13 chemotype has remained constant, in part supports the predicted C13 binding model in the Casp6 putative allosteric pocket. Modifications at the dihydro-benzofuran end have highlighted the importance of the shape of the pocket delimited by L200. Similarly, a change at the other end of the C13 molecule indicates that the furan ring is likely involved in critical binding interactions as predicted by modelling. It is necessary to assess additional analogues to validate the model, especially in light of the fact that alanine mutagenesis of two Casp6 allosteric pocket residues, L200 and K284, which are predicted to make key interactions with C13 according to our *in silico* model, did not abolish Casp6 inhibition by C13. Analogues bearing amide and carboxy isosteres, as well as various linkers attaching both ends and the central phenyl ring would be critical to verify the importance of these functionalities for hydrogen bonding or lipophilic interactions. In addition, the fact that Casp6 putative allosteric pocket is mainly composed of flexible loops, complicates accurate predictions of ligand binding using computational tools. Hence, additional C13 *in silico* binding models have to be experimentally evaluated and methods such as X-ray crystallography could be used to validate C13 binding to the postulated Casp6 allosteric site.

The identification of S10G and Casp6 potential interaction sites using H/DX-MS was inconclusive. S10G contains a pyrimidinetrione PAINS structure^53^, and may act as a promiscuous compound, hence, one should consider H/DX-MS results with caution. The observed S10G potency for Casp6, however, is not entirely dependent on the presence of pyrimidinetrione structure since the S10P analogue lacking this structure still inhibited Casp6.

This study illustrates challenges faced in the early stage drug discovery and serves as a starting point for the future optimisation of our identified hits towards potent Casp6 allosteric inhibitors.

## Methods

### Identification of Casp6 variants

Casp6 missense variants were identified from the dbSNP short genetic variation NCBI database (https://www.ncbi.nlm.nih.gov/projects/SNP/snp_ref.cgi?geneId=839).

### Compounds

Compounds were purchased from ChemBridge Corporation (San Diego, CA, USA), Sigma-Aldrich (St. Louis, MO, USA) TimTec Inc (Newark, DE, USA), and Vitas-M Laboratory Ltd (Champaign, IL, USA), and solubilised in 100% DMSO at 10 mM stock concentration.

### Plasmids

Plasmid pET23b-Casp6-His^63^ encoding full-length human Casp6 (Addgene plasmid 11823) was distributed by Dr. Guy Salvesen (Sanford Burnham Prebys Medical Discovery Institute, CA, USA). The pET23b-Casp6(WT)p20/p10-His plasmid^36^ for *E. coli* codon optimized co-expression of Casp6-WT large and small subunits and pET23b( + ) plasmid encoding Casp6-C163A^8^ were previously described.

### Cloning

Single mutations were introduced into pET23b-Casp6-His plasmid using QuikChange Site Directed Mutagenesis Kit (Stratagene, La Jolla, CA, USA) with the following primers and corresponding reverse sequence: A34E: 5′-AAA AGA GAA ATG TTT GAT CCG GAA GAA AAG TAC AAA ATG GAC CAC-3′, E35K: 5′-GAG AAA TGT TTG ATC CGG CAA AAA AGT ACA AAA TGG ACC AC-3′, A109T: 5′-TGT CAA CTG TTA GCC ACA CAG ATG CCG ATT GCT TT-3′, T182S: 5′-GGA TGT AGT AGA TAA TCA GTC AGA GAA GTT GGA CAC CAA-3′, L200A: 5′-CCG TTT ACA CGG CCC CTG CTG GAG C-3′, K284A: 5′-AAT GCT AAC TGC CAA GCT GCA TTT C-3′. Mutations were introduced into pET23b-Casp6(WT)p20/p10-His plasmid with the following primers and corresponding reverse sequence: L200A: 5′-GTT TAC ACC GCG CCG GCG GGC-3′, K284A: 5′-CAT GCT GAC CGC GAA ACT GCA CT TC-3′. Plasmids were verified by Sanger sequencing (McGill University and Genome Quebec Innovation Center, Montreal, QC, CA).

### Protein purification

Plasmids encoding full-length Casp6-WT and variants, large and small subunits of Casp6-WT and variants, and Casp6-C163A were expressed in *E. coli* BL21(DE3)pLysS strain (Promega, Fitchburg, WI, USA) and purified^51^. Purity was evaluated by SDS-PAGE and Coomassie blue staining, and protein concentration was measured using Quick Start Bradford 1x Dye Reagent (Bio-Rad Laboratories).

### Casp6 active site titration and activity assays

Casp6 active sites were quantified using zVAD-FMK (N-benzyloxycarbonyl-Val-Ala-Asp-(O-methyl)-fluoromethylketone, MP Biomedicals, Santa Ana, CA, USA)^51,64^. Casp6 activity was measured in Stennicke’s buffer (SB: 20 mM piperazine-N,N′-bis-(2-ethanesulfonic acid) pH 7.2, 100 mM NaCl, 10 mM DTT, 1 mM EDTA, 0.1% 3-[(3-cholamidopropyl)-dimethylammonio]-2-hydroxy-1-propanesulfonic acid, 10% sucrose)^65^ using 40 µM Ac-VEID-AFC (Ac-Val-Glu-Ile-Asp-7-Amino-4-trifluoromethylcoumarin, Enzo Life Sciences, Farmingdale, NY, USA)^64^ and 10-250 nM active site titrated Casp6. Reactions were initiated by Ac-VEID-AFC addition and followed for 20 min at 37 °C in a black clear bottom 96-well microplate (Costar, Corning, NY, USA) in Synergy H4 Hybrid instrument (BioTek, Winooski, VT, USA) with excitation/emission at 380/505 nm. Released AFC was calculated from AFC standard curve (0-25 µM). Casp6 VEIDase activity (released pmol AFC/min) was calculated from the initial linear reaction part. Ac-VEID-AFC hydrolysis initial velocities were measured with 10 nM active-site titrated Casp6 and 1–300 nM Ac-VEID-AFC. Initial velocities v versus substrate concentration [S] were fit to a rectangular hyperbola using Michaelis-Menten equation in GraphPad Prism 5 (GraphPad Software, La Jolla, CA, USA).

### Western blot analyses

25 ng purified Casp6 were separated by SDS-PAGE on 15% gel, transferred to polyvinylidene fluoride membrane in Trans-Blot Turbo Transfer system (Bio-Rad Laboratories) and probed with 1:10,000 rabbit polyclonal neoepitope antiserum 10630 against active human Casp6 ^174^PLDVVD^179^ p20 subunit^10^, 1:250 mouse monoclonal IgG1 clone B93-4 against human Casp6 ^271^GKKQVPCFASMLTKK^285^ p10 subunit (BD Biosciences, San Jose, CA, USA), and 1:1000 mouse monoclonal IgG1 SC81653 antibody against human pro-Casp6 24–293 aa (Santa Cruz Biotechnology, Dallas, TX, USA). Antibodies were diluted in Tris-buffered saline with 0.1% Tween-20 and 5% non-fat dry milk. Proteins were detected with 1:5000 secondary anti-mouse (Jackson ImmunoResearch Laboratories, West Grove, PA, USA) and anti-rabbit (Dako, Glostrup, Denmark) antibodies conjugated to horseradish peroxidase using ECL western blotting detection reagent (GE Healthcare), and Carestream Biomax MR films (Kodak, Rochester, NY, USA).

### Crystallisation and structure determination

Fully processed Casp6 mutants E35K and A109T were crystallised in 100 mM sodium acetate pH 4.6 with 2 M sodium chloride. Crystals were grown by hanging drop vapour diffusion at 22 °C and were flash-frozen. X-ray diffraction data was collected at 100 K on beamline F1 with an ADSC Q-270 detector at the Cornell High Energy Synchrotron Source (MacCHESS). Data was processed by HKL2000^66^. The structures were solved by molecular replacement with Phaser^67^ in Phenix^68^ using the crystal structure of wild-type Casp6 (PDB: 4FXO) as a search model, and re-built manually in Coot^69^. Refinement was carried out by phenix.refine^70^, with non-crystallographic symmetry restraints applied for the A109T mutant structure. Crystallographic data collection and structure refinement statistics are presented in Supplementary Table S2. Structural images were prepared with PyMOL (The PyMOL Molecular Graphics System, Version 1.3 Schrödinger, LLC).

### Molecular modelling

The MOE package (Molecular Operating Environment (MOE), 201; Chemical Computing Group ULC, 1010 Sherbooke St. West, Suite #910, Montreal, QC, Canada, H3A 2R7) was used for structural analysis, protein protonation (Protonate 3D), automated active site prediction (Site Finder module, defaults parameters) and hit compounds binding mode analysis. The ConSurf server was used for analysing sequence conservation of the members of human caspase family, as well as the mapping of this information onto the Casp6 crystal structure^71^. Molecular graphics were rendered with PyMOL (Version 1.7.1).

### Virtual screening by molecular docking

The Sigma Myriascreen II collection (10,000 compounds) and the Chembridge Premium Set (50,000 compounds) were acquired from their commercial provider (www.sigmaaldrich.com and www.chembridge.com, respectively). In-house script was used to filter out compounds with undesired substructures. The retained 57,681 compounds were canonicalized and their three-dimensional coordinates were generated using Chemaxon Standardizer, JChem 5.1.2 (www.chemaxon.com). Protonation at pH 7.4 was performed on Ac-VEID-AFC-bound Casp6-WT structure (PDB: 3OD5) using the Protonate 3D module in MOE with the Amber12:EHT forcefield. The histidine H41 that lines the allosteric site was attributed the HSD protonation state. The AutoDockTools interface^72^ was used to prepare the Ac-VEID-AFC-bound Casp6-WT protonated structure into PDQT format. The docking grid was defined as a 16 × 16 × 22-Å box around the entire putative allosteric site when docking the compounds library as well as the follow up C13 analogues. Although the C13 compound purchased and tested is a racemic mixture (presence of a chiral centre at the dihydrobenzofuran branching point), the model derived from docking strongly suggests the R-form to be most likely the isomer to optimally bind in the putative site. For blind docking of S10G compound (Fig. 5c) the whole Casp6 structure was considered for the docking search space, also using the PDB entry 3OD5 but in which the peptide substrate was removed. Docking was carried out with Autodock Vina^73^ using the default 9 output poses per compound docked. Autodock Vina scoring function was used to rank compounds by lowest energy and the top-1% ranked candidates were visualy inspected using PyMOL and the MOE package.

### *In vitro* screening of compounds

In fluorescence-based Casp6 activity assay, 10 µM Ac-VEID-AFC was added to 20 nM Casp6 in SB with 100 μM compound and 1% dimethyl sulfoxide (DMSO), and reaction rate was measured for 20 min at 37 °C as above. Compound potency was calculated by normalising Ac-VEID-AFC cleavage rate to 1% DMSO control. In colorimetric Casp6 activity assay, 200 µM Ac-VEID-*p*NA (N-acetyl-Val-Glu-Ile-Asp-p-nitroanilide, Enzo Life Sciences) was added to 200 nM Casp6 in SB with 100 μM compound, and Ac-VEID-*p*NA cleavage rate was measured at 37 °C for 30 min in clear 96-well plate (Sartstedt, Nümbrecht, Germany) using Synergy H4 plate reader at 405 nm. Absorbance was converted to the released *p*NA amount based on a standard curve of 0–250 µM *p*NA. Cleavage rates were calculated from the linear assay phase and were normalised to 1% DMSO control. In Casp6-C163A cleavage assay, 0.5 ng/µl Casp6-WT was preincubated with 100 µM inhibitor, 5 µM Q-VD-OPh or 1% DMSO in SB (30 min, RT), followed by transfer on ice, addition of 0.5 µg/µl Casp6-C163A, and incubation (37 °C, 5, 10 and 15 min). Reactions were stopped by adding SDS-PAGE sample buffer (62.5 mM Tris pH 6.8, 2% SDS, 0.1 M DTT, 0.01% Bromphenol Blue, 10% glycerol) and heating (5 min, 95 °C). Samples (2 µl) were analysed on 15% SDS-PAGE gels, proteins were stained with Coomassie blue, and gels were de-stained with 10% acetic acid. The intensities of protein bands were quantified from scanned gels using ImageJ. The band intensity of Casp6-C163A without a prodomain was plotted against the time and the Casp6-C163A cleavage rates were calculated from the slopes of linear plots. The % activity of Casp6-WT in the presence of inhibitors was calculated relative to DMSO control.

### IC_50_ determination

IC_50_ values were determined against 20 nM Casp6 active sites in fluorescence-based Casp6 activity assay with 10 µM Ac-VEID-AFC and 1 nM-500 µM inhibitor. IC_50_ was calculated with GraphPad Prism 5 by a nonlinear regression fit for log inhibitor vs relative Casp6 activity (% DMSO control) using a Hill slope of – 1, and top and bottom constraints of 100 and 0, respectively.

### Determination of inhibition mechanism

Compounds, S10G (2.5 µM, 4.2 µM, 10 µM), and C13 (5 µM, 14 µM, 50 µM), were added to 20 nM active site titrated Casp6 and enzyme activity was measured with eight different Ac-VEID-AFC substrate concentrations (5.0 μM–200 μM) using fluorescence-based Casp6 activity assay as above. The apparent *K*_M_ and V_max_ values were determined through nonlinear regression analysis of Michaelis-Menten plots (v vs. [S]) using GraphPad Prism 5. The inhibition mode was determined from the pattern of converging Lineweaver-Burk plots (1/v vs. 1/[S])^54^. The dissociation constant (*K*_i_) for Casp6-inhibitor complex was determined from the x-axis intercept (which is equal to −*K*_i_) in the plot of Lineweaver-Burk slopes vs. inhibitor concentration^74^. Alternatively, inhibition model and *K*_i_ were determined by global fit of kinetic data to the competitive, noncompetitive, noncompetitive (mixed) or uncompetitive inhibition models using respective equations in GraphPad Prism 5. IC_50_ curves for S10G and C13 against Casp6 were generated using the fluorescence-based activity assay with 5–75 µM Ac-VEID-AFC.

### Bioinformatic analyses

Multiple sequence alignments were generated with Clustal Omega tool (http://www.ebi.ac.uk/Tools/msa/clustalo/) using UniProt accession numbers for human caspases: P29466, P42575, P42574, P49662, P51878, P55212, P55210, Q14790, P55211, Q92851, Q6UXS9, P31944, and Casp6 from different species: P55212, K7B3Z2, Q3T0P5, S9XS51, A0A0B8S0B0, O35397, O08738, L5K505, S7N3D7, A0A0Q4ALF8, O93415, A0A0Q3PLE0, Q9IB66, V9L2A1, Q9I8S9, Q52KK6, G0XQH6, Q9NHF9, K1R808.

### Hydrogen/deuterium exchange combined with mass spectrometry (HDX-MS)

HDX-MS was carried out as described^75^. HDX was initiated by diluting 25 µM Casp6 stock solution with or without S10G (125 µM) into the D_2_O-based buffer (20 mM Tris-HCl, 120 mM NaCl, 2 mM DTT, pD 8.5, based on pD = pH_read_ + 0.40)^76^ using 1:9 dilution ratio. For Casp6 in the presence of S10G, stock solution of Casp6 with S10G was diluted by D_2_O-based buffer containing S10G. The HDX incubation period and temperature were set to 10, 60, 900, 2400 s and 25 °C, respectively. HDX was quenched with chilled quenching buffer (300 mM glycine, 8 M urea in H_2_O, pH 2.4) using 1:2 dilution ratio. Quenched solutions were flash frozen in MeOH containing dry ice, and samples were stored at −80 °C until use. For the undeuterated control, initial dilution was made in H_2_O buffer. Prior to ultra-high performance liquid chromatography (UHPLC)-MS analysis, the deuterated Casp6 was digested in an on-line immobilised pepsin column prepared in house. Resulting peptides were loaded onto a C_18_ analytical column (1-mm inner diameter, 50 mm length; Thermo Fisher Scientific, Waltham, MA, USA) equipped to an Agilent 1290 Infinity II UHPLC system. Peptides for each sample were separated using a 5–40% linear gradient of acetonitrile containing 0.1% formic acid for 8 min at a 65 µl/min flow rate. To minimize back-exchange, the columns, solvent delivery lines, injector and other accessories were placed in an ice bath. The C_18_ column was directly connected to the electrospray ionization source of the LTQ Orbitrap XL (Thermo Fisher Scientific), and mass spectra of peptides were acquired in positive-ion mode for *m*/*z* 200–2000. Duplicate measurements were performed for each time point. Identification of peptides was carried out in separate experiments by tandem MS (MS/MS) analysis in data-dependent acquisition mode, using collision-induced dissociation. All MS/MS spectra were analysed using Proteome Discoverer 1.4 (Thermo Fisher Scientific). Peptide searching results were further manually inspected, and only those verifiable were used in HDX analysis. The deuteration (%) as a function of incubation time was determined using HDExaminer 2.3 (Sierra Analytics, Modesto, CA). The first two amino acid residues in peptides were excluded from the analysis.

## Supplementary information

Supplementary information file

## Data Availability

All generated or analysed study data are included in this published article and its Supplementary Information file.
