# Supplementary material for: Identification of Allosteric Inhibitors against Active Caspase-6
Source: Sci Rep. 2019 Apr 2;9:5504. doi: 10.1038/s41598-019-41930-7 (PMC6445123; doi:10.1038/s41598-019-41930-7)
Supplement: Supplementary file 1 — Supplementary information file [file 41598_2019_41930_MOESM1_ESM.pdf]

Identification of Allosteric Inhibitors against Active Caspase-6

Agne Tubeleviciute-Aydin*[a,b], Alexandre Beautrait*[c], Jeffrey Lynham[a,d], Gyanesh Sharma[a,b], Alexei Gorelik[e], Ludovic J. Deny[c], Naoto Soya[f], Gergely L. Lukacs[f], Bhushan Nagar[e], Anne Marinier[c], Andrea C. LeBlanc[a,b,d]

[a] Bloomfield Center for Research in Aging, Lady Davis Institute for Medical Research, Jewish General Hospital, 3755 Ch. Cote Ste-Catherine, Montreal, Quebec, Canada H3T 1E2

[b] Department of Neurology and Neurosurgery, McGill University, 3775 University St., Montreal, Quebec, Canada H3A 2B4

[c] Institute for Research in Immunology and Cancer, Université de Montréal, 2590, chemin de Polytechnique, Montreal, Quebec, Canada H3T 1J4

[d] Department of Anatomy and Cell Biology, McGill University, 3640 University St., Montreal, Quebec, Canada H3A 0C7

[e] Department of Biochemistry, McGill University, 3649 promenade Sir-William-Osler, Montreal, Quebec, Canada H3G 0B1

[f] Department of Physiology and Biochemistry, McGill University, 3655 Promenade Sir-William-Osler, Montréal, Québec, Canada H3G 1Y6

*These authors contributed equally to this work.

Corresponding author: Andrea LeBlanc, PhD, Bloomfield Center for Research in Aging, Lady Davis Institute for Medical Research, Sir Mortimer B Davis Jewish General Hospital, 3755 ch. Côte Ste-Catherine, Montréal, QC, Canada H3T 1E2. Tel.: +1 (514) 340 8222 ext 24976. Fax.: +1 (514) 340 8295. e-mail address: andrea.leblanc@mcgill.ca

Supplementary Figure S1.

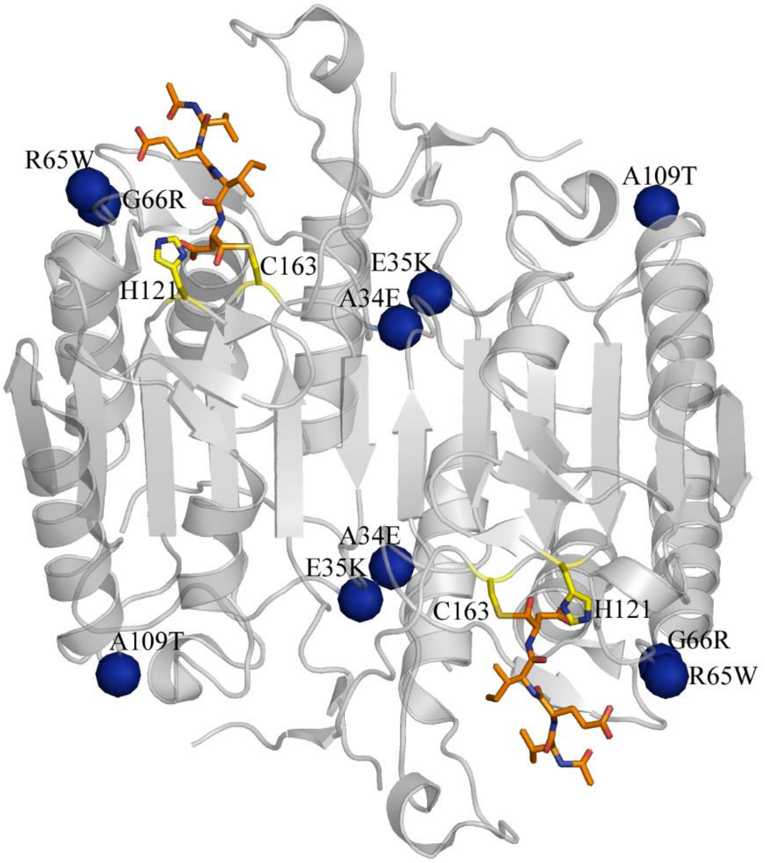

Supplementary Figure S1. The location of Casp6 amino acid substitutions encoded by rare non-synonymous missense single nucleotide polymorphisms (SNPs) investigated in this study (A34E, E35K, A109T, T182S) and our previous work (R65W, G66R)[1]. Amino acid substitutions are indicated by blue spheres on a fully mature active site liganded dimeric Casp6 structure (PDB: 3OD5). Catalytic Casp6 residues (C163 and H121) are shown in yellow sticks and active site-bound Ac-VEID-CHO is depicted in orange sticks. T182S (not shown) is located in the intersubunit linker, which is removed upon Casp6 proteolytic maturation.

Supplementary Figure S2.

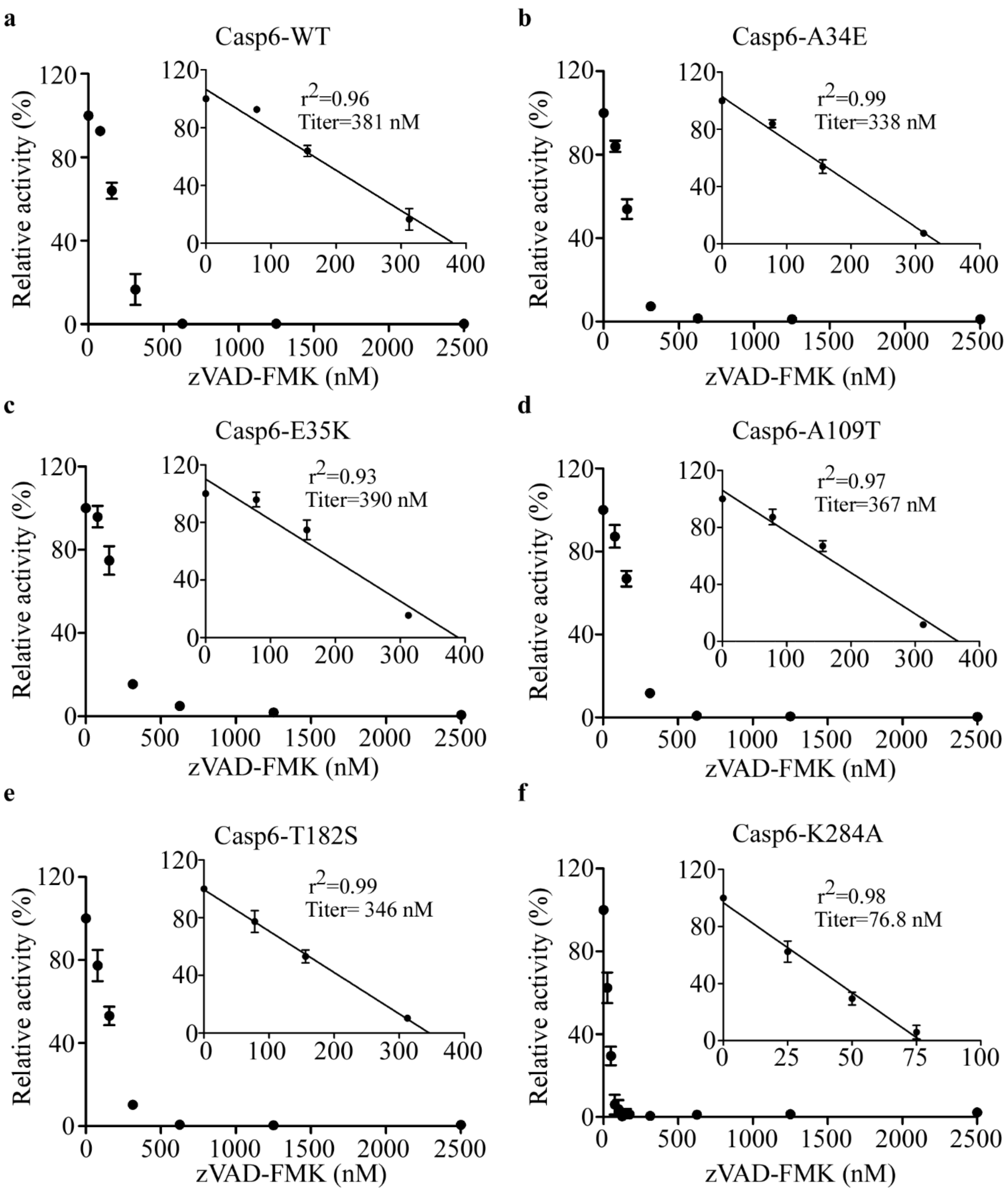

Supplementary Figure S2 (continued).

g

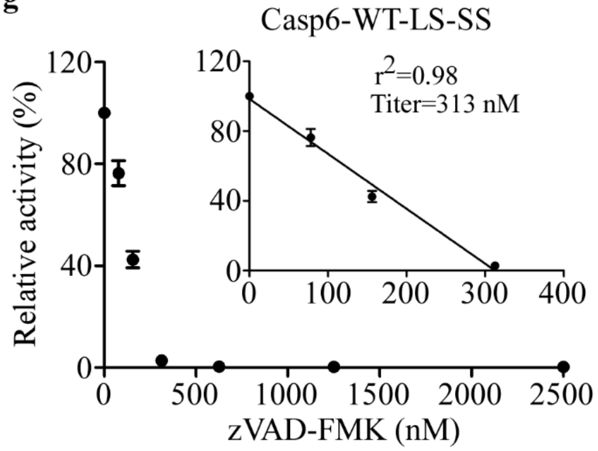

h

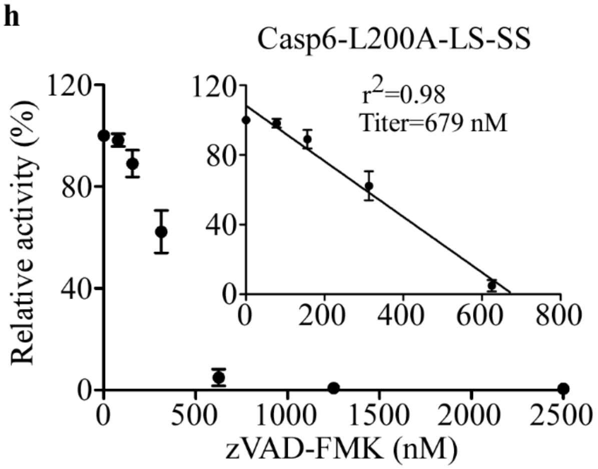

i

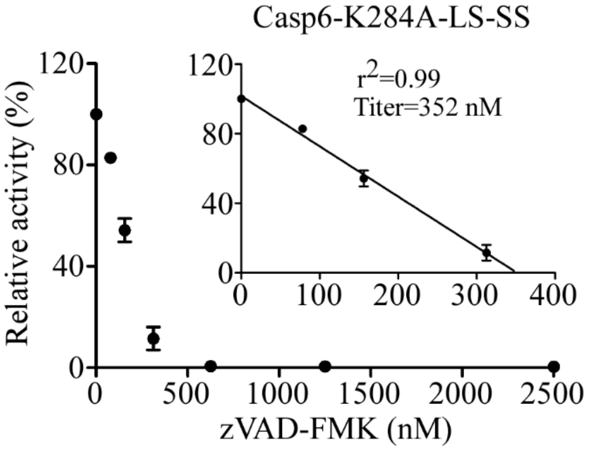

Supplementary Figure S2. Active site titration of purified recombinant Casp6 variants. Active site titration of ~380 nM recombinant Casp6-WT (**a**), Casp6-A34E (**b**), Casp6-E35K (**c**), Casp6-A109T (**d**), Casp6-T182S (**e**), Casp6-K284A (**f**), Casp6-WT-LS-SS (**g**), Casp6-L200A-LS-SS (**h**), and Casp6-K284A-LS-SS (**i**) with irreversible inhibitor zVAD-FMK. The inlets of the graphs show linear regression of data points corresponding to zVAD-FMK concentrations at which Casp6 inhibition is less than 100%, the x-axis intercept (titer) is equal to the active caspase concentration in the titration assay. The data points represent mean ± standard deviation (SD) from three independent measurements. The recombinant purified Casp6 samples are 82-102% active, except for Casp6-K284A and Casp6-L200A-LS-SS preparations, which are 20% and 178% active, respectively.

Supplementary Figure S3.

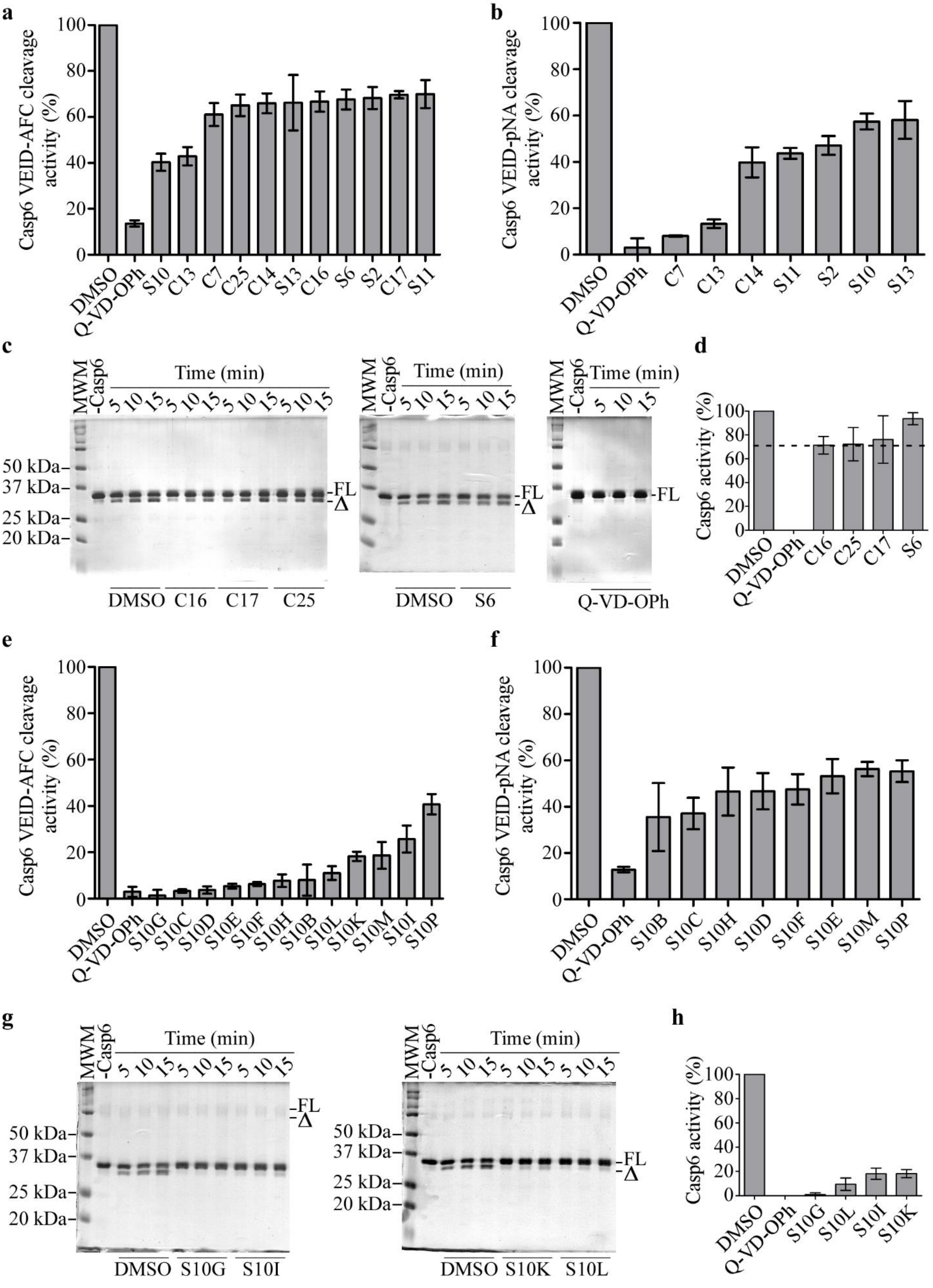

Supplementary Figure S3. Screening of hits identified through the *in silico* screen and their commercially available analogues. (a) Ac-VEID-AFC cleavage activity of recombinant Casp6 in the presence of 100 µM of compounds identified through the Sigma (S) and Chembridge (C) libraries or 5 µM Q-VD-OPh pan-caspase inhibitor relative to DMSO control. (**b**) Ac-VEID-pNA cleavage activity of recombinant Casp6 in the presence of 100 µM of small molecule inhibitors or 5 µM Q-VD-OPh relative to DMSO control. (**c**) Coomassie stained gels and **(d)** quantification of the rate of Casp6-C163A prodomain cleavage by active Casp6 in the presence of 100 µM of small molecule inhibitors or 5 µM Q-VD-OPh relative to DMSO control, FL – full-length Casp6-C163A, Δ – Casp6-C163A without a prodomain. A broken line indicates 70% Casp6 activity cut-off. **(e)** Ac-VEID-AFC cleavage activity of recombinant Casp6 in the presence of 100 µM of S10 analogues or 5 µM Q-VD-OPh relative to DMSO control. **(f)** Ac-VEID-pNA cleavage activity of recombinant Casp6 in the presence of 100 µM of S10 analogues or 5 µM Q-VD-OPh relative to DMSO control. (**g**) Coomassie stained gels and **(h)** quantification of the rate of Casp6-C163A prodomain cleavage by active Casp6 in the presence of 100 µM of S10 analogues or 5 µM Q-VD-OPh relative to DMSO control, FL – full-length Casp6-C163A, Δ – Casp6-C163A without a prodomain.

Supplementary Figure S4.

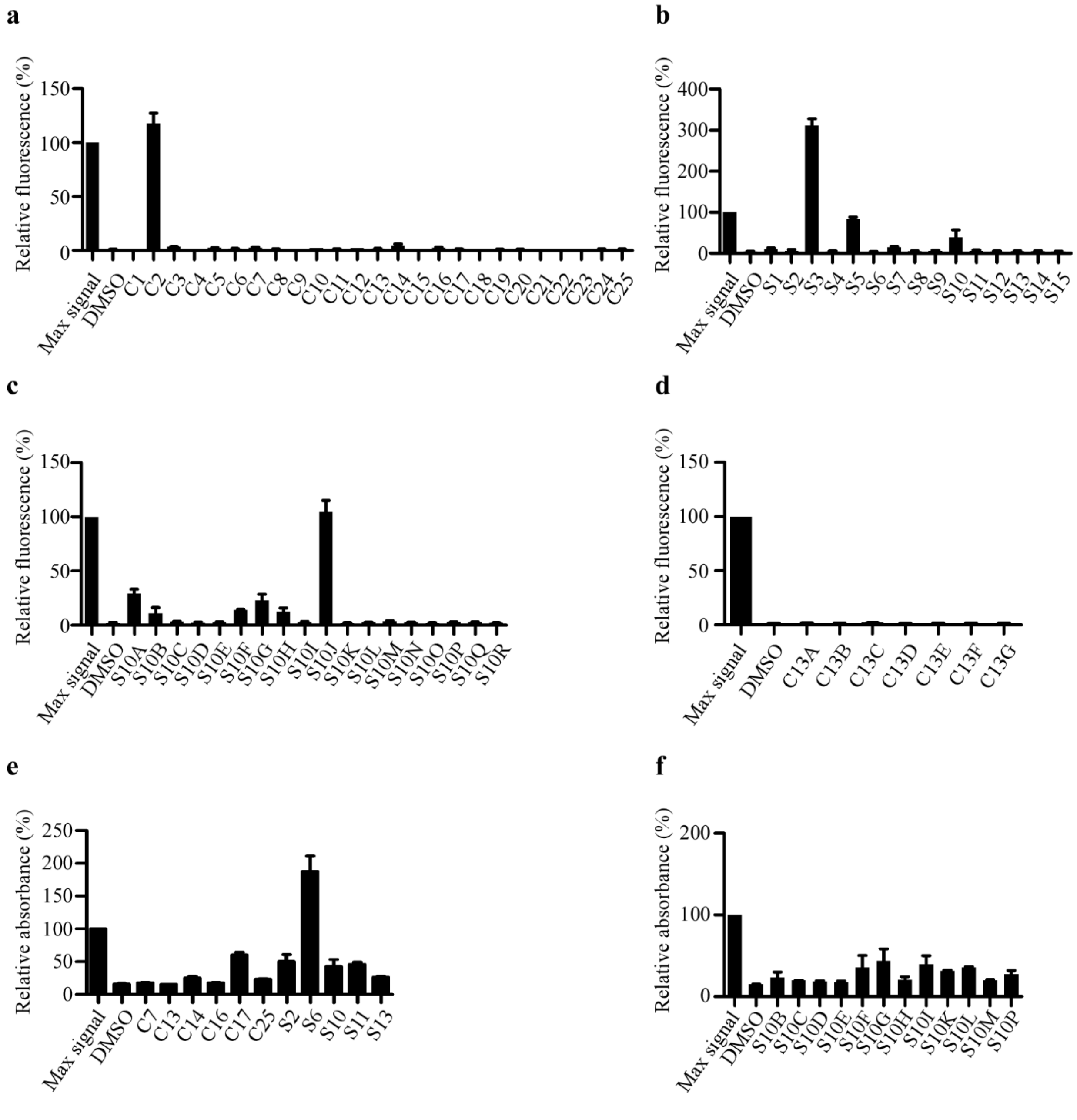

Supplementary Figure S4. Intrinsic fluorescence and absorbance of compounds. Relative fluorescence of (**a**) Chembridge library compounds, (**b**) Sigma library compounds, (**c**) S10 analogues, and (**d**) C13 analogues in Stennicke's buffer at 100 µM compound concentration measured at an excitation wavelength of 380 nm and an emission wavelength of 505 nm. Max signal was set to 100%, and it corresponds to a maximal fluorescence of AFC released by Casp6 in the absence of inhibitors under screening assay conditions. Relative absorbance of hits from Chembridge and Sigma libraries (**e**), and S10 analogues (**f**) in Stennicke's buffer at 100 µM compound concentration measured at a wavelength of 405 nm. Max signal was set to 100%, and it corresponds to a maximal absorbance of

p-nitroanilide (*p*NA) released by Casp6 in the absence of inhibitors under screening assay conditions. All readings were measured in duplicate.

Supplementary Figure S5.

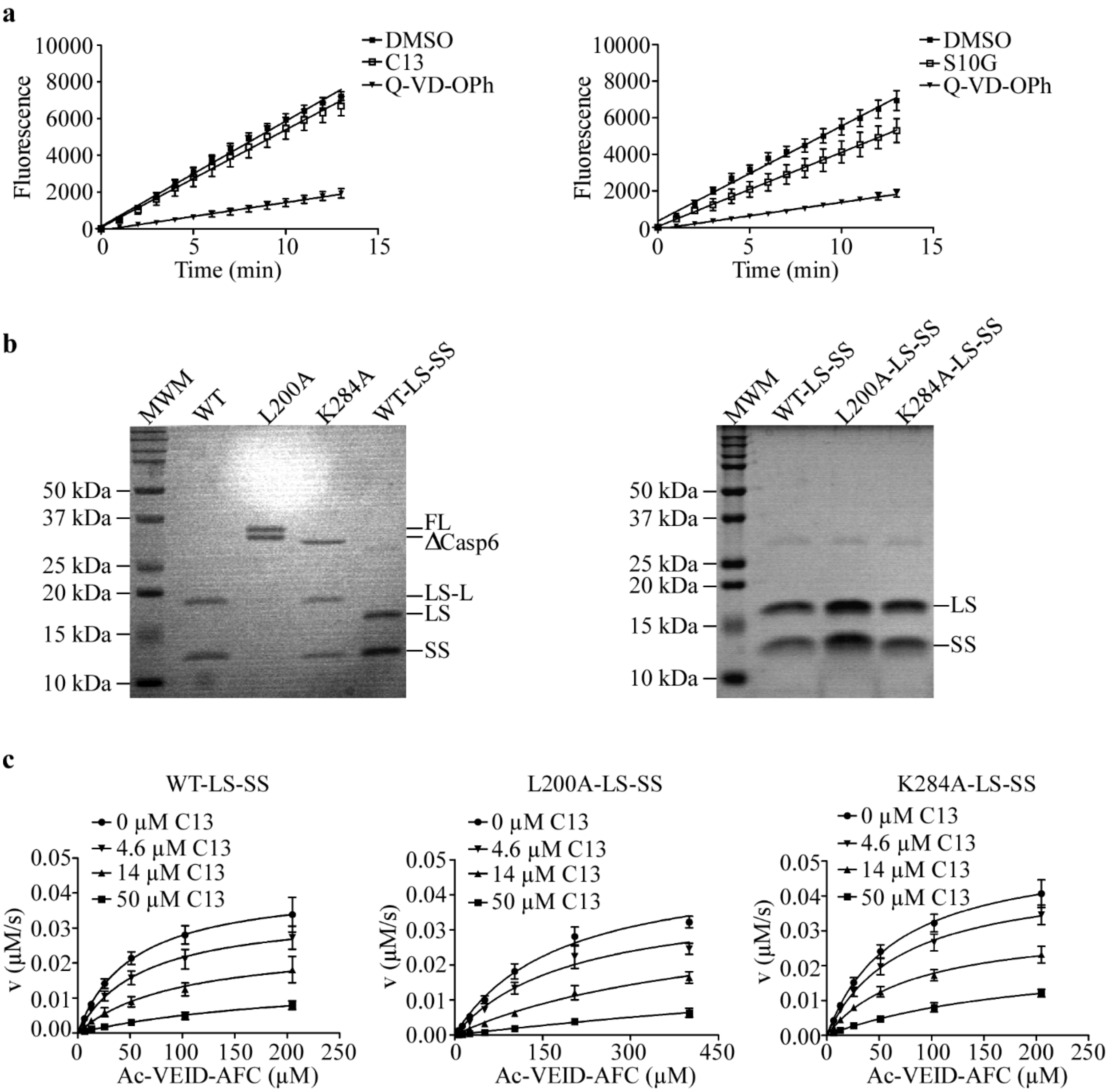

WT-LS-SS

C13 (μM)	K_M (μM)	V_{max} (μM s^{-1})
0	52.3 ± 7.6	0.043 ± 0.007
4.6	61.3 ± 4.9	0.035 ± 0.004
14	96.6 ± 19.9	0.026 ± 0.007
50	242.4 ± 67.4	0.017 ± 0.003

L200A-LS-SS

C13 (μM)	K_M (μM)	V_{max} (μM s^{-1})
0	190.3 ± 54.0	0.050 ± 0.005
4.6	201.1 ± 80.3	0.039 ± 0.006
14	371.4 ± 41.1	0.032 ± 0.004
50	593.2 ± 190.0	0.014 ± 0.002

K284A-LS-SS

C13 (μM)	K_M (μM)	V_{max} (μM s^{-1})
0	65.3 ± 4.4	0.054 ± 0.006
4.6	70.5 ± 6.0	0.046 ± 0.003
14	80.7 ± 6.5	0.032 ± 0.003
50	218.6 ± 55.5	0.025 ± 0.002

Supplementary Figure S5. Mechanism of Casp6 inhibition with C13 and S10G. (**a**) Inhibition of Casp6 with C13 (left panel) and S10G (right panel) is reversible. The recovery of Casp6 activity after 60 min pre-incubation with C13 and S10G at a concentration of inhibitor equal to 10-fold the IC_{50} value followed by a rapid 100-fold

dilution into Casp6 activity assay solution. The control samples DMSO and Q-VD-OPh were pre-incubated and diluted in the absence of inhibitor, and in the presence of an irreversible inhibitor Q-VD-OPh, respectively. Data points represent mean ± standard error of the mean (SEM) from three independent experiments. **(b)** Coomassie stain of SDS-PAGE of purified recombinant WT-, L200A-, and K284A-Casp6 (left panel) expressed from a full-length Casp6 encoding construct (Fig. 1c), and copurified LS and SS of WT-, L200A-, and K284A-LS-SS-Casp6 (right panel). FL (full-length Casp6), ΔCasp6 (Casp6 without a prodomain), LS-L (large subunit with linker), LS (large subunit), and SS (small subunit) depict different bands of Casp6. (**c**) Michaelis-Menten kinetic plots (top panel) and calculated K_M and V_{max} values (bottom panel) for Casp6-WT-LS-SS (left panel), Casp6-L200A-LS-SS (middle panel), and Casp6-K284A-LS-SS (right panel) enzymatic activity in presence of 1% DMSO (0 µM C13) or increasing doses of C13. Data represent mean ± SD from three independent experiments.

Supplementary Figure S6.

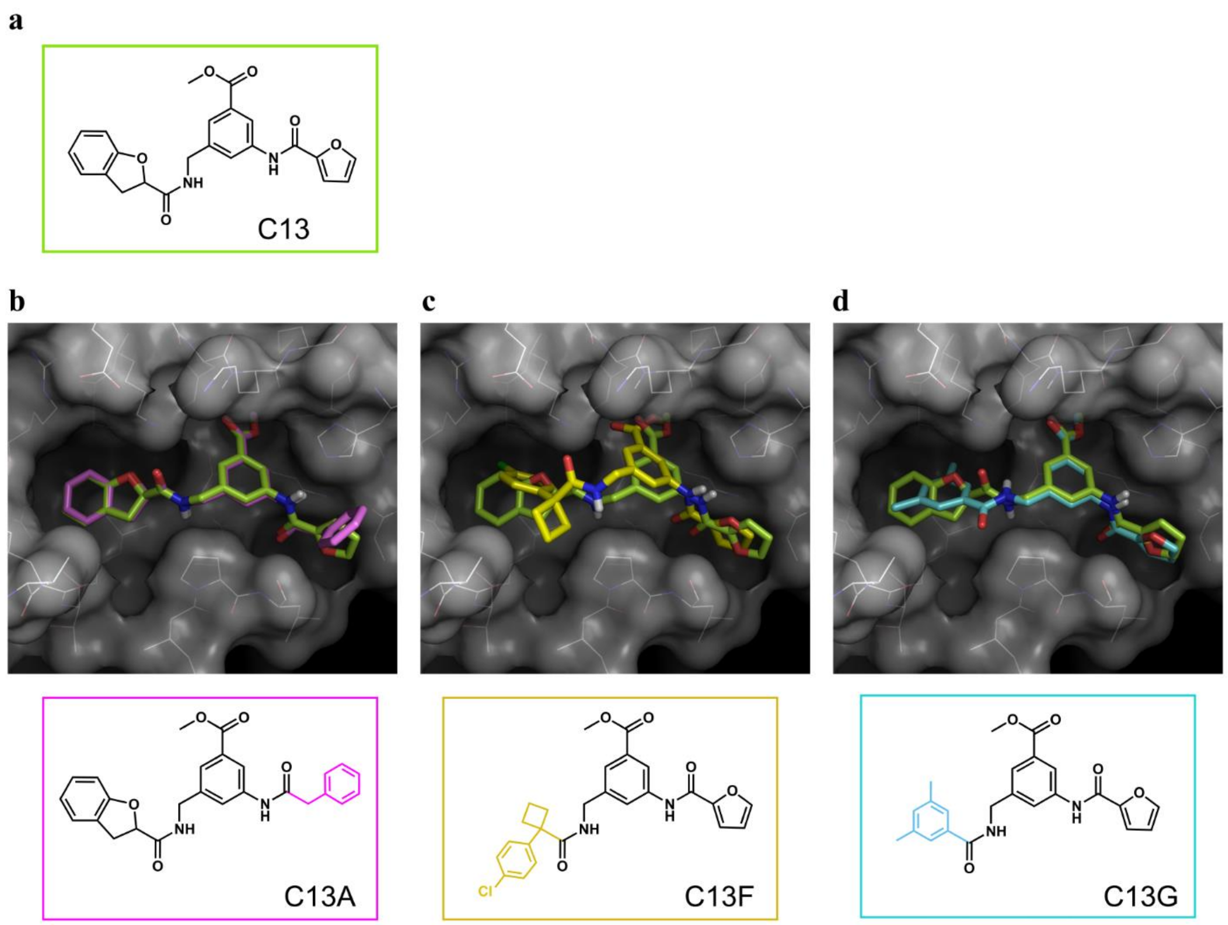

Supplementary Figure S6. **Predicted docking poses of selected C13 analogues in the Casp6 allosteric pocket.** (**a**) C13 two-dimensional structure. Among the seven analogues studied, a reasonable binding model was obtained for 3 compounds only: (**b**) C13A, pink, (**c**) C13F, yellow, (**d**) C13G, cyan. Compounds are in sticks representation and overlaid with C13 docking pose (green). The Casp6 is represented by a transparent surface, and residues from the putative allosteric pocket are depicted in white lines. The Casp6 3OD5 structure was used to generate the figure.

Supplementary Figure S7.

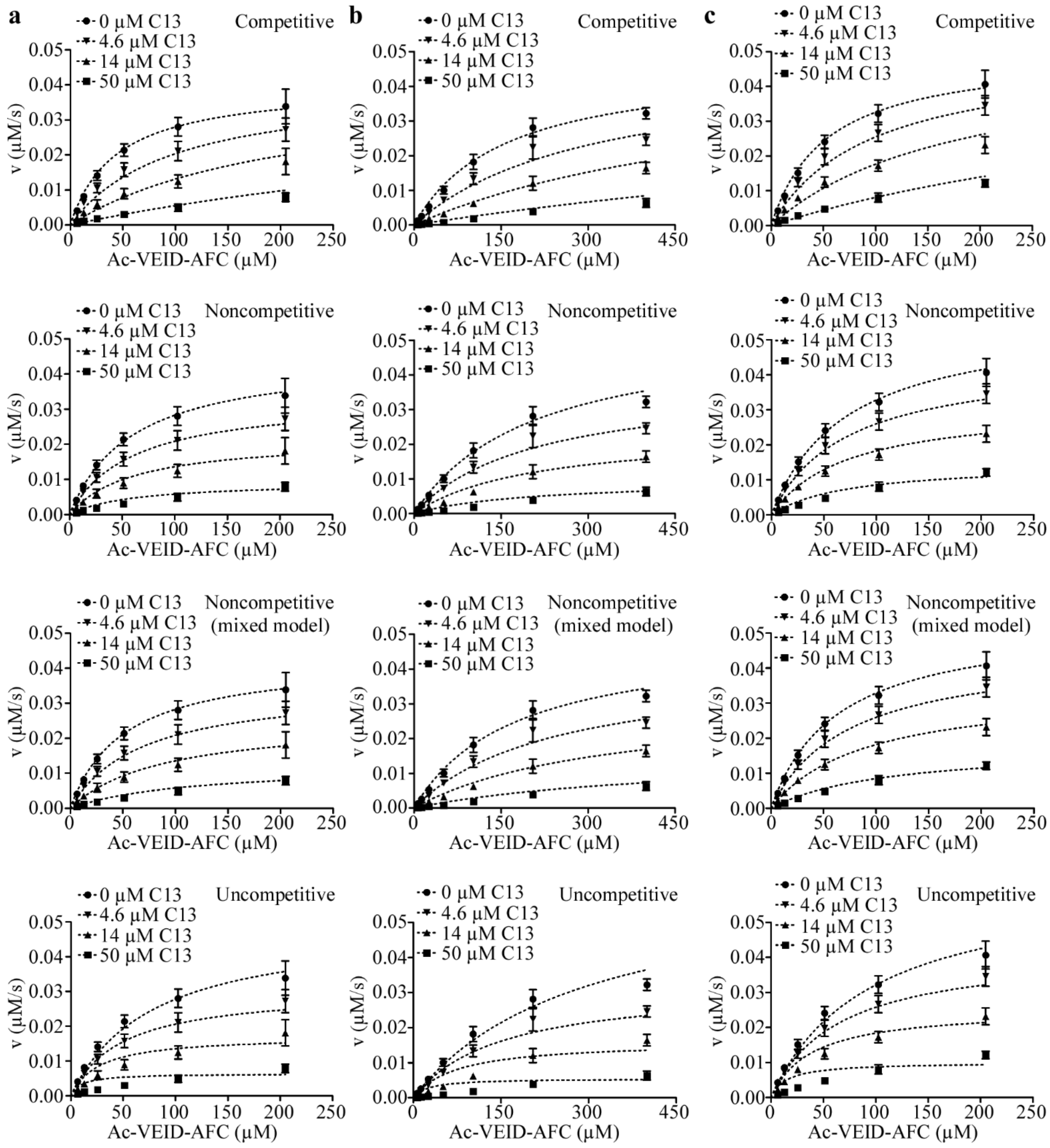

d

	Global Fit R^2 values			
	Competitive	Noncompetitive	Noncompetitive (mixed model)	Uncompetitive
Casp6-WT-LS-SS	0.9534	0.9606	0.9635	0.9335
Casp6-L200A-LS-SS	0.9669	0.9671	0.9699	0.9353
Casp6-K284A-LS-SS	0.9694	0.9780	0.9794	0.9553

Supplementary Figure S7. Inhibition mechanism of C13. Kinetic data of Ac-VEID-AFC hydrolysis by Casp6-WT-LS-SS (**a**), Casp6-L200A-LS-SS (**b**), and Casp6-K284A-LS-SS (**c**) in the presence of 0-50 μM C13 was globally fitted to the competitive, noncompetitive, noncompetitive (mixed) or uncompetitive inhibition models. Data points represent the average of three independent experiments ± SD. (**d**) The R^2 values of the global fit for each inhibition model.

Supplementary Figure S8.

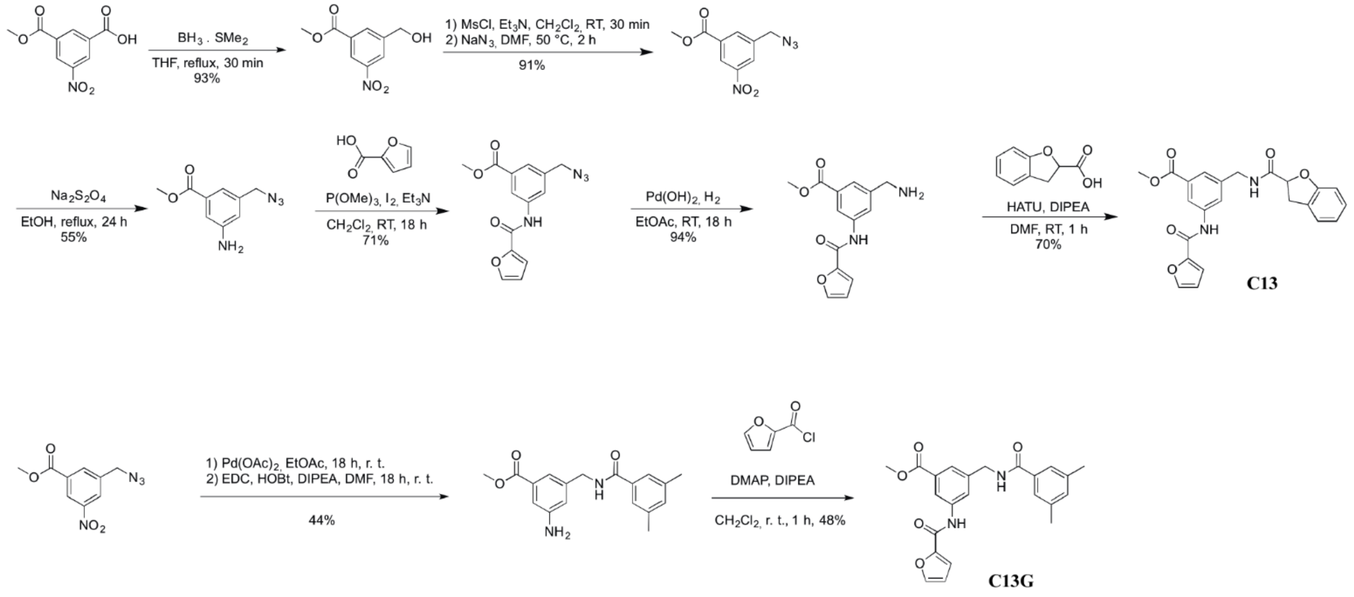

Supplementary Figure S8. Scheme for synthesis of C13 and C13G compounds.

Supplementary Figure S9.

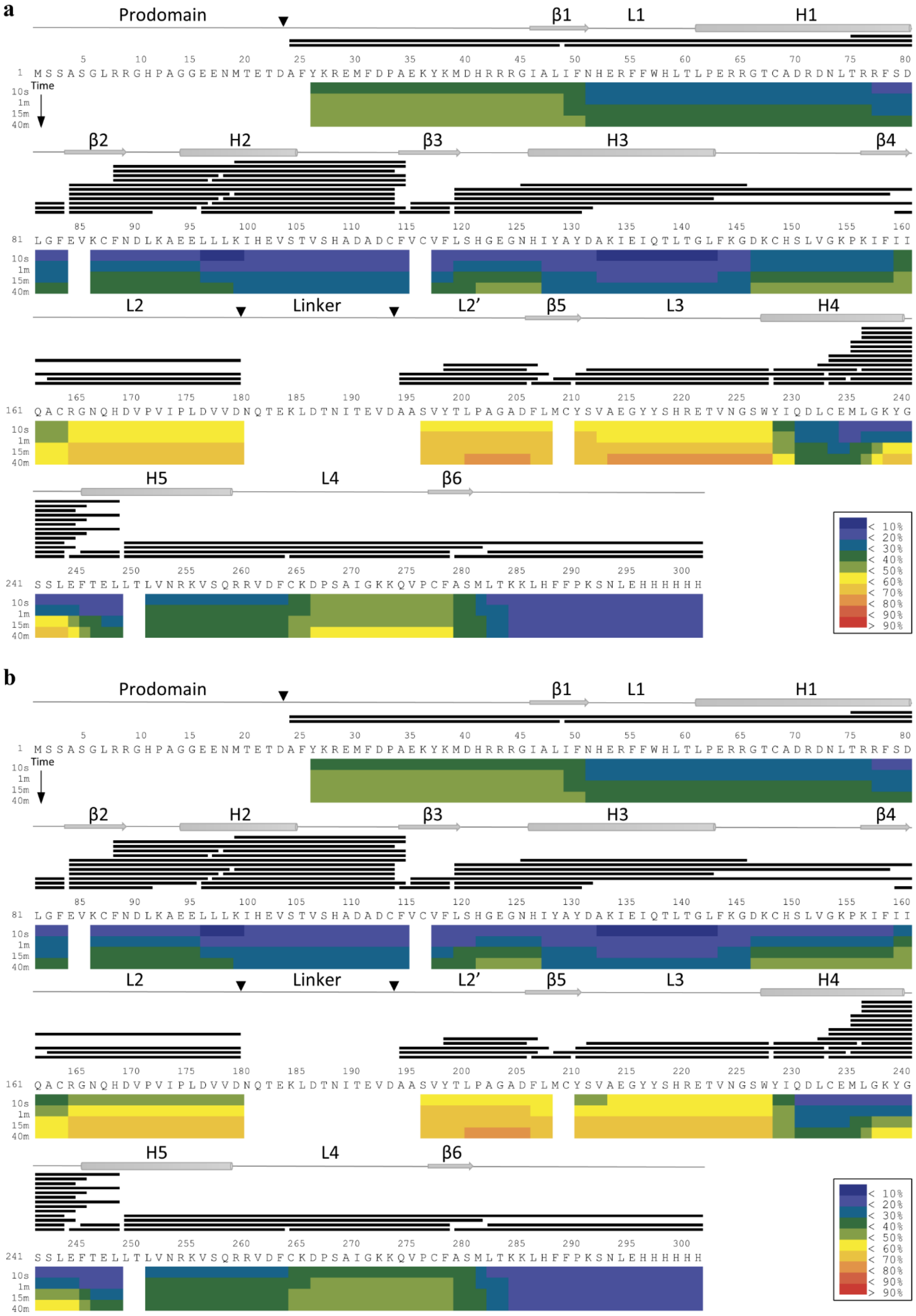

Supplementary Figure S9. H/D exchange heat map of the relative deuterium uptake. The percent relative deuterium level for each H/D exchange incubation time (10 s, 1 min, 15 min, and 40 min) is mapped onto Casp6 linear sequence for each peptic peptide of Casp6 incubated without S10G (**a**) and with S10G (**b**). Peptic peptide coverage map is indicated by the black bars above the primary sequence. Secondary structure elements, loops (L), alpha-helices (H) and beta-strands (β) are depicted above the peptic peptide coverage map and are derived from the apo mature structure of Casp6 (PDB: 2WDP). Pro-Casp6 processing sites are indicated by black triangles. The percent relative deuterium uptake was calculated as a ratio of observed deuterium uptake and the theoretical maximum deuterium uptake for each peptide. Fully processed Casp6-WT-LS-SS, lacking both the prodomain (1-23 aa) and linker (180-193 aa) and containing LEHHHHHH C-terminal tag was used in the H/DX-MS experiments. The percent relative deuterium uptake of each peptic peptide represents the average of three independent experiments.

Supplementary Figure S10.

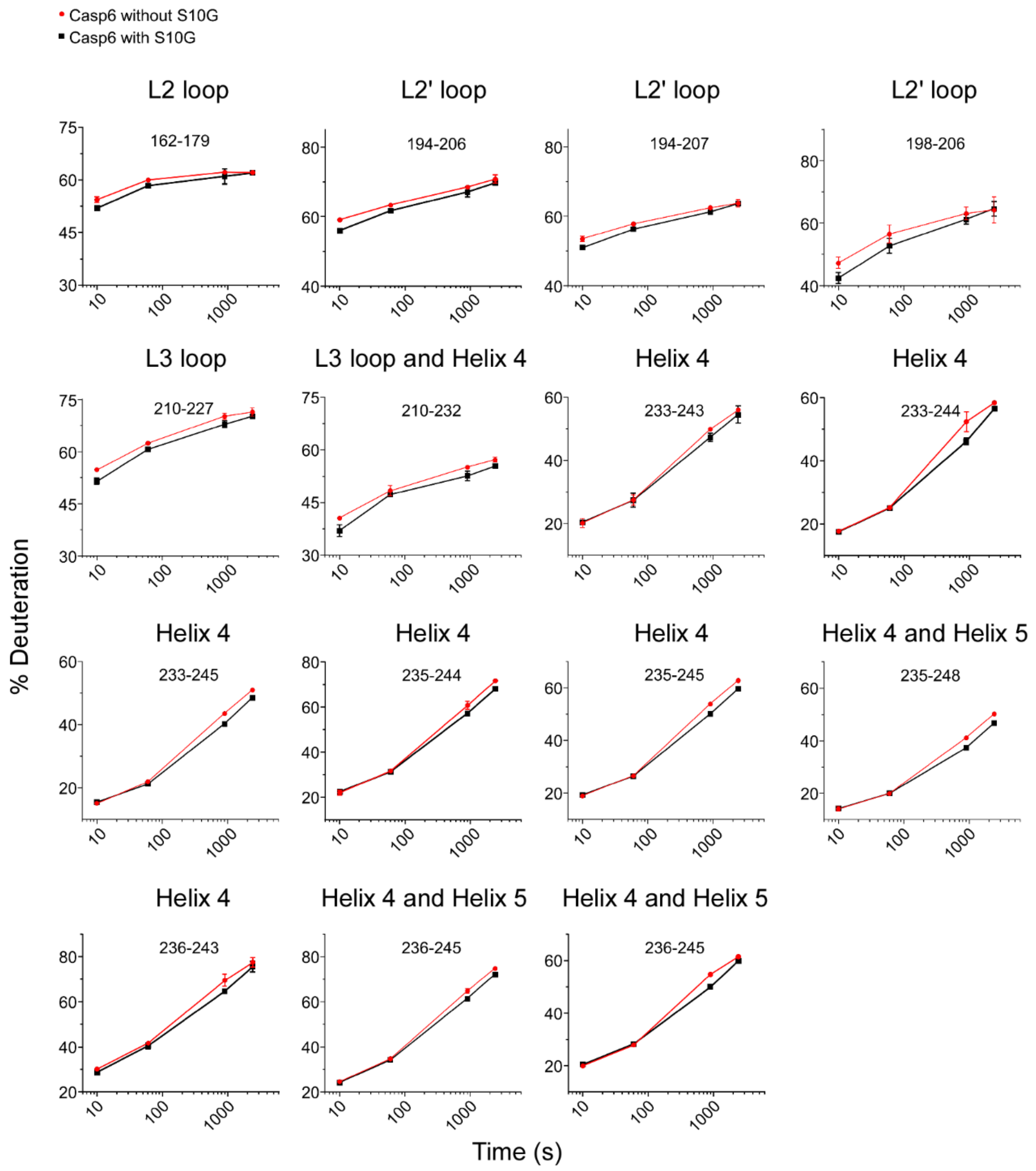

Supplementary Figure S10. Relative deuterium uptake plots comparing the Casp6 in the absence (red) and presence (black) of S10G as a function of H/D exchange incubation time. Data points represent mean ± SD from three independent experiments. Amino acid numbering same as in Fig. S9.

Supplementary Figure S11.

a

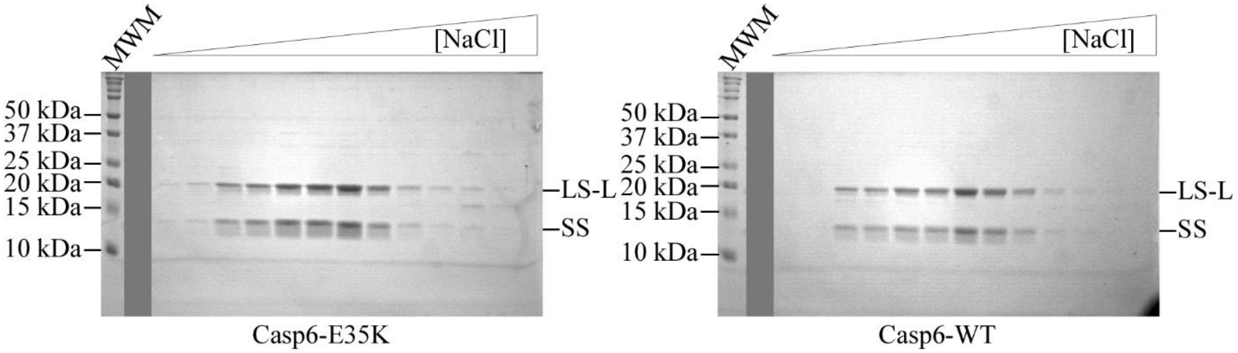

b

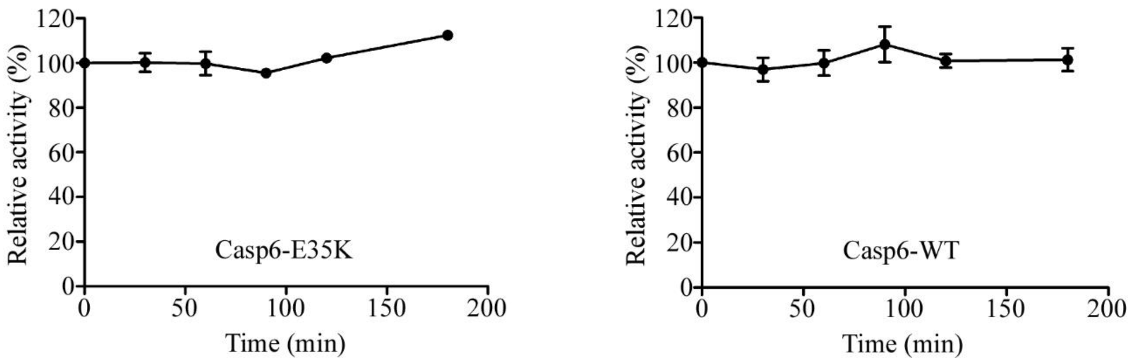

Supplementary Figure S11. (**a**) Coomassie-stained gel of Casp6-E35K (left panel) and Casp6-WT (right panel) fractions eluted from anion exchange column with NaCl gradient. Co-elution of Casp6 LS-L and SS subunits suggests that LS-L and SS remain associated during anion exchange purification step. (**b**) *In vitro* stability of purified recombinant Casp6-E35K (left panel) and Casp6-WT (right panel) in Stennicke's buffer at 37 ºC was determined by measuring caspase residual activity against Ac-VEID-AFC substrate as a function of time. Enzyme activity at the time point 0 was set to 100%.

Supplementary Figure S12.

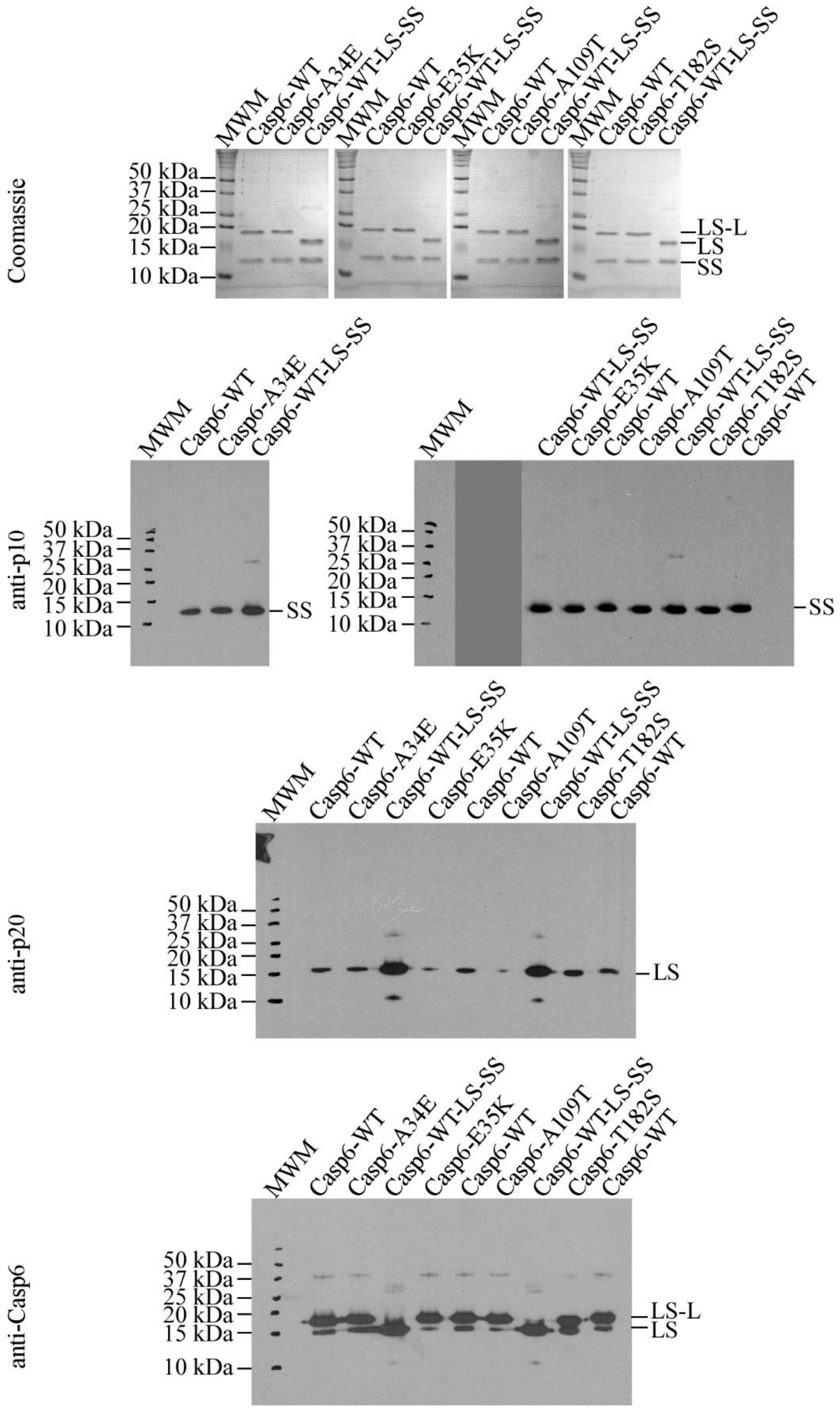

Supplementary Figure S12. Full-length gels (top panel) and western blots (three bottom panels) of figure 1d.

Supplementary Table S1. Rare Casp6 SNPs investigated in this study.

SNP ID	Minor Allele Frequency (MAF)	Amino acid change
rs11574696	NA	A34E
rs11574697	NA	E35K
rs5030674	T=0.0021/252[a]	A109T
rs5030593	A=0.0023/280[a]	T182S

[a]MAF from ExAC database

NA – not available

Supplementary Table S2. Crystallographic data collection and refinement statistics.

	Caspase-6 E35K	Caspase-6 A109T
PDB code	6DEV	6DEU
Data collection		
Space group	P 2_1	P 2_1 2_1 2
Unit cell a, b, c (Å)	64.39, 91.33, 86.68	91.11, 63.93, 86.53
Unit cell α, β, γ (°)	90, 91.80, 90	90, 90, 90
Wavelength (Å)	0.92	0.92
Resolution range (Å)	50 – 2.35 (2.43 – 2.35)	50 – 2.80 (2.90 – 2.80)
Total reflections	298,470 (19,228)	187,761 (18,566)
Unique reflections	41,219 (3,496)	13,030 (1,263)
Multiplicity	7.2 (5.5)	14.4 (14.7)
Completeness (%)	98 (83)	100 (100)
R_{meas} (%)	11 (> 100)	11 (>100)
R_{pim} (%)	5 (87)	4 (>100)
Mean I/σ(I)	15.1 (1.0)	23.9 (1.0)
$CC_{1/2}$	(0.43)	(0.67)
Wilson B factor (Å^2)	32.8	44.9
Refinement		
Protein copies per ASU	4	2
Resolution range (Å)	30.35 – 2.35 (2.43 – 2.35)	37.10 – 2.80 (2.90 – 2.80)
Reflections used	35,268 (466)	11,597 (422)
Reflections for R_{free}	2,018 (32)	1,155 (44)
R_{work} (%)	20.5 (27.2)	26.7 (39.1)
R_{free} (%)	24.9 (41.5)	31.2 (40.9)
Non-hydrogen atoms	7,425	3,600
protein	7,240	3,588
water	185	12
Average B factor (Å^2)	45.5	61.4
protein	45.8	61.5
water	36.2	36.2

	Caspase-6 E35K	Caspase-6 A109T
RMSD bond lengths (Å)	0.002	0.004
RMSD bond angles (°)	0.47	0.67
Ramachandran favored (%)	97.0	95.9
Ramachandran allowed (%)	2.9	3.9
Ramachandran outliers (%)	0.1	0.2
Rotamer outliers (%)	0.5	1.0
Clashscore	1.7	7.0

Values in parentheses are for the highest-resolution shell. ASU, asymmetric unit. RMSD, root-mean-square deviation.

Supplementary Table S3. Structures and acquisition numbers of compounds from Sigma library purchased from commercial sources.

Compound	Structure	Acquisition number
S1	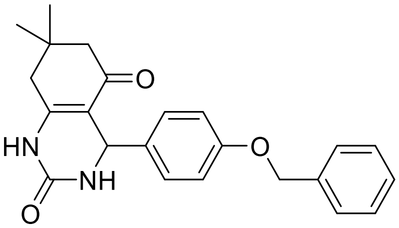	L134716[a]
S2	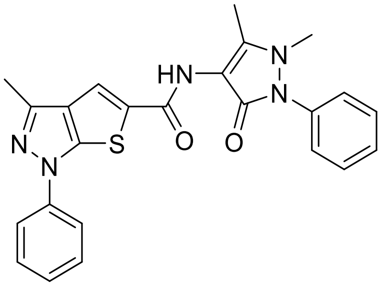	L344575[a]
S3	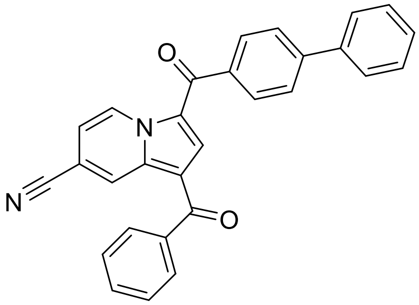	R872172[a]
S4	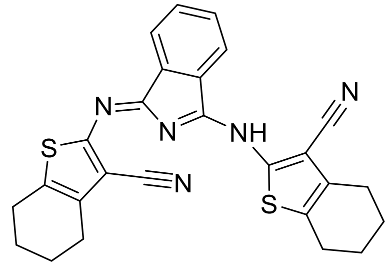	MFCD02170893[a]
S5	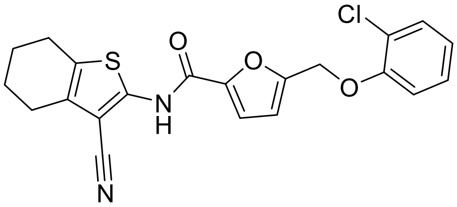	7503517[b]
S6	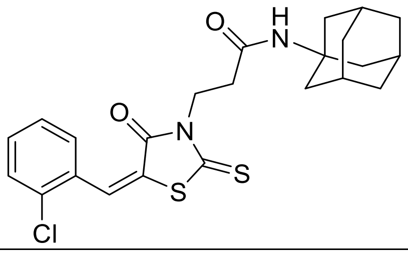	ST029443[c]

Compound	Structure	Acquisition number
S7	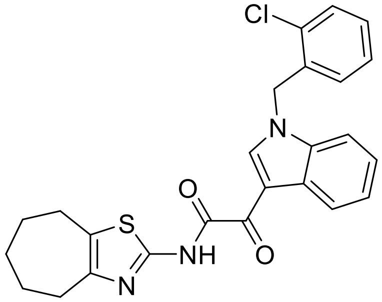	ST072073[c]
S8	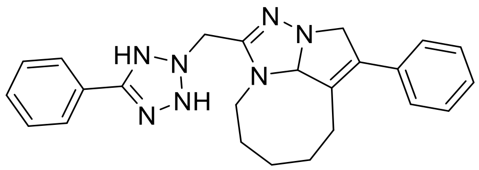	STK533677[d]
S9	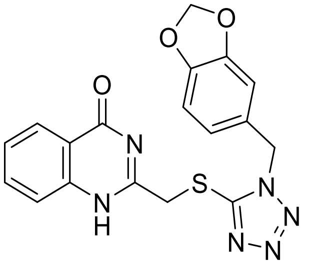	STK690188[d]
S10	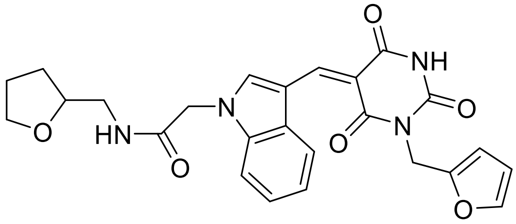	STK846193[d]
S11	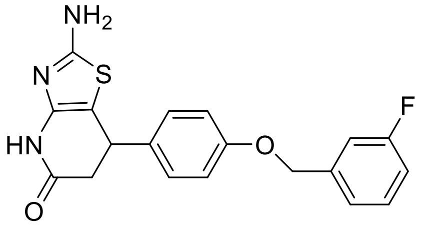	STK691500[d]
S12	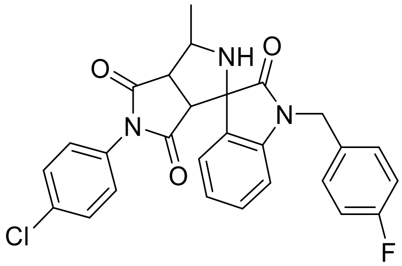	STK777008[d]
S13	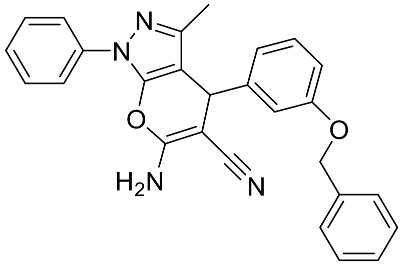	STK748610[d]

Compound	Structure	Acquisition number
S14	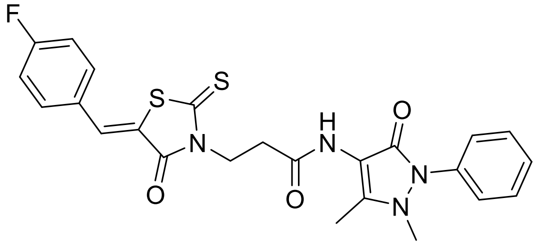	STL297810[d]
S15	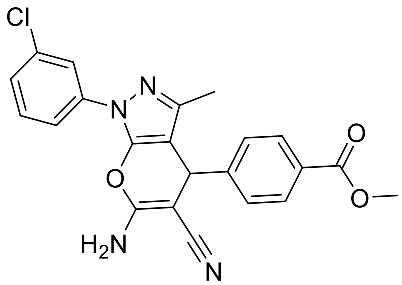	STK051741[d]

[a]Sigma-Aldrich, [b]ChemBridge, [c]TimTec, [d]Vitas-M Laboratory.

Supplementary Table S4. Structures and acquisition numbers of compounds from Chembridge library purchased from ChemBridge company.

Compound	Structure	Acquisition number
C1	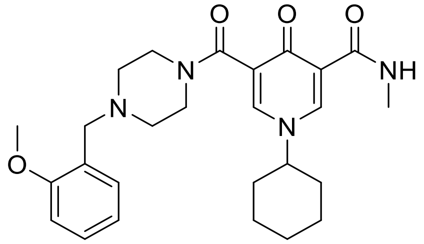	11616733
C2	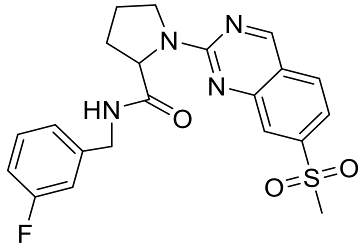	16373584
C3	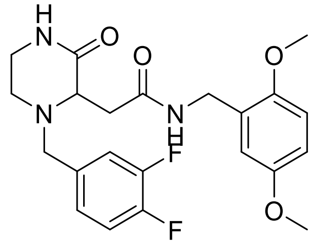	20829437
C4	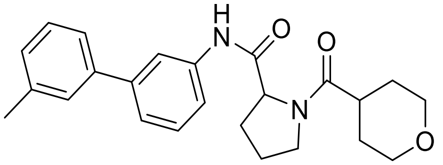	23107276
C5	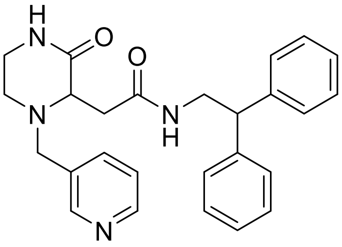	27358942
C6	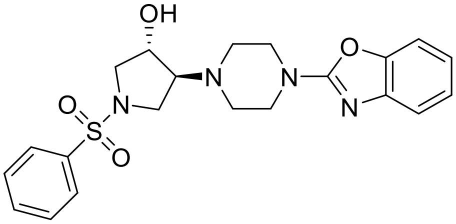	44031268

Compound	Structure	Acquisition number
C7	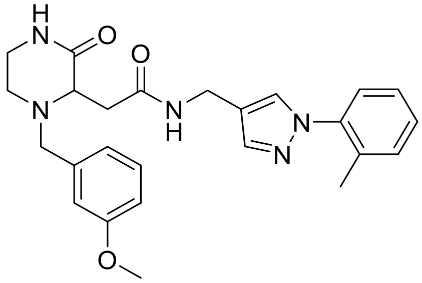	50865496
C8	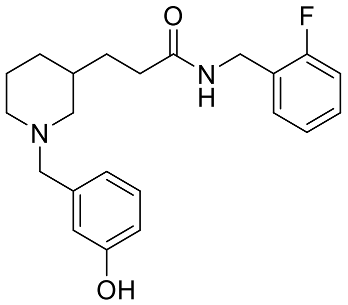	53636868
C9	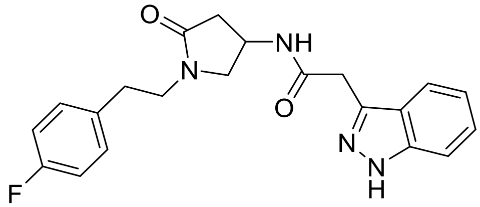	54022613
C10	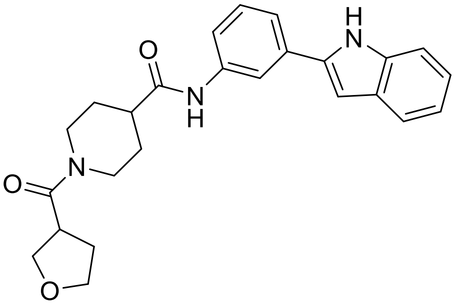	58437674
C11	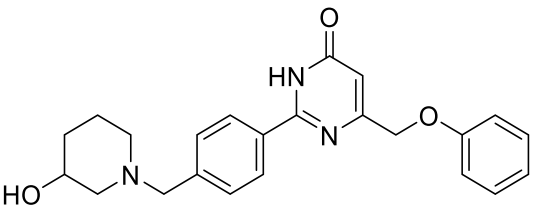	65791511
C12	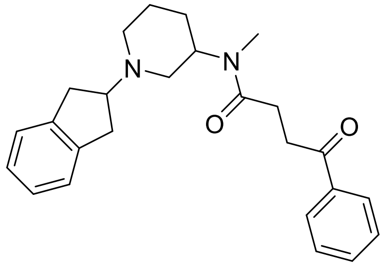	67929114

Compound	Structure	Acquisition number
C13	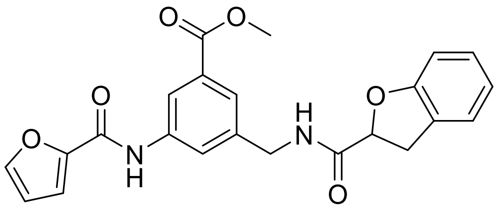	78056422
C14	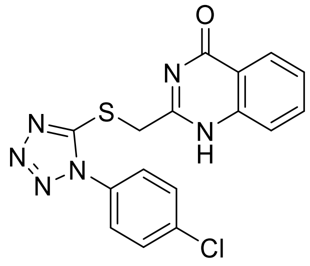	7992796
C15	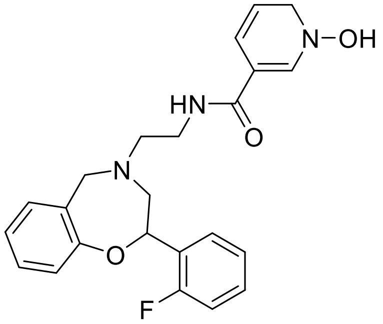	87713315
C16	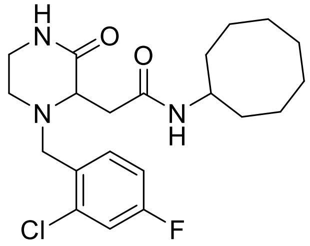	90704693
C17	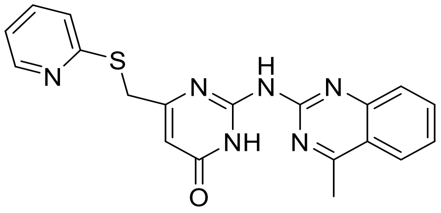	9125383
C18	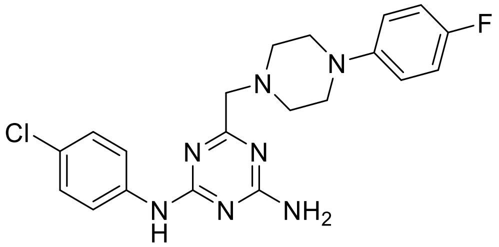	9129750
C19	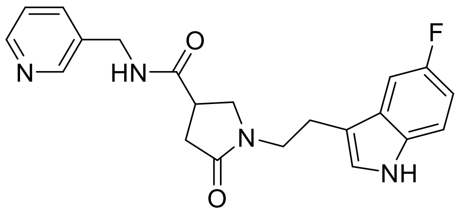	9132146

Compound	Structure	Acquisition number
C20	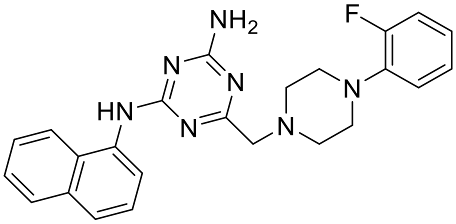	9138900
C21	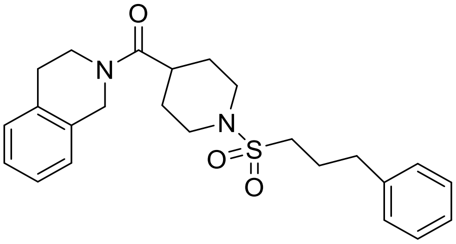	9142622
C22	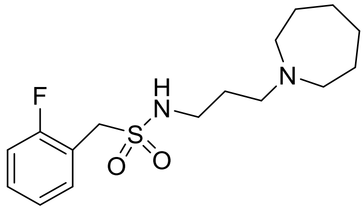	9148556
C23	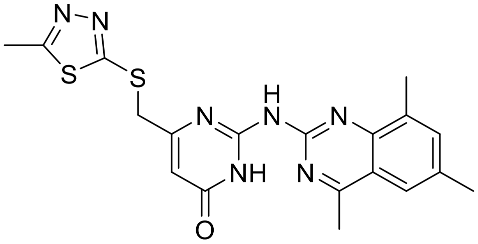	9149567
C24	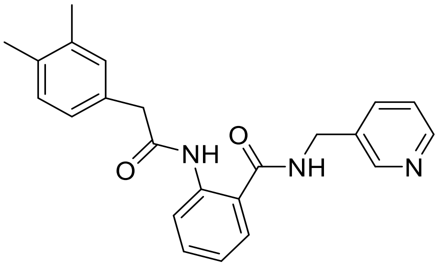	9152306
C25	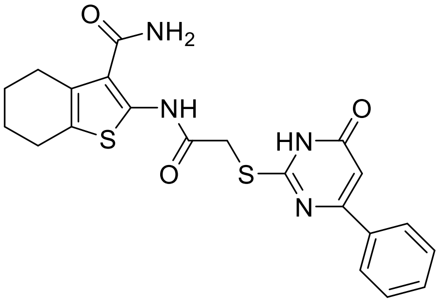	9155407

Supplementary Table S5. Acquisition numbers of S10 and C13 analogues purchased from ChemBridge company.

Compound	Acquisition number
S10B	6368769
S10C	6872009
S10D	6872306
S10E	6873308
S10F	6874457
S10G	6876815
S10H	6877515
S10I	7373957
S10K	7416656
S10L	7437371
S10M	7465425
S10P	7916479
C13A	29715502
C13B	73645252
C13C	21798626
C13D	13634649
C13E	70234979
C13F	62049676
C13G	72715584

METHODS

Chemistry

Unless otherwise noted, all common reagents and solvents were obtained from commercial suppliers and used without further purification. All reactions requiring anhydrous conditions were conducted under a positive pressure of nitrogen. 1H NMR were recorded on a 400 MHz or a 300 MHz Bruker instrument, in $CDCl_3$ or $SO(CD_3)_2$ (DMSO-d6) as solvent, with chemical shifts (δ) referenced to internal standard $CDCl_3$ (7.26 ppm) or $SO(CD_3)_2$ (2.50 ppm). LC-MS were recorded on an Agilent Technologies Model 6120 quadrupole. Abbreviations: THF: tetrahydrofuran, AcOH: acetic acid, EtOAc: ethyl acetate, HATU: (1-[Bis(dimethylamino)methylene]-1H-1,2,3-triazolo[4,5-b]pyridinium 3-oxide hexafluorophosphate, DMF: N,N-dimethylformamide, DIPEA: diisopropylethylamine, DCM: dichloromethane, DMAP: dimethylaminopyridine.

Methyl 3-(hydroxymethyl)-5-nitrobenzoate. According to method[2], 3-(Methoxycarbonyl)-5-nitrobenzoic acid (5 g, 22.21 mmol) was dissolved in dry THF (15.0 mL) at room temperature. The colourless solution was stirred and cooled to -15 °C when borane-methyl sulfide complex (20 mL, 40.0 mmol) was added dropwise over 30 min. The resulting solution was allowed to warm up to room temperature and refluxed for 30 min. At 0 °C, AcOH/water (1/1, 5 mL) was added carefully. The mixture was allowed to warm up to room temperature, neutralised with saturated aqueous $NaHCO_3$ (150 mL) and extracted with EtOAc (3 x 50 mL). The combined organic layers were washed with water (50 mL), brine (2 x 50 mL), dried over anhydrous Na_2SO_4, filtered and concentrated to give a yellow oil. The crude material was adsorbed on celite and purified by automated chromatography (EtOAc in hexanes) to give methyl 3-(hydroxymethyl)-5-nitrobenzoate as a white solid (4.36 g, 20.65 mmol, 93%). Spectral data were consistent with a previous report[3]. 1H NMR (DMSO-d6, 400 MHz) δ (ppm) 8.51 (t, 1H, J = 1.5 Hz), 8.42 (s, 1H), 8.32 (s, 1H), 5.68 (t, 1H, J = 4.4 Hz), 4.70 (d, 2H, J = 4.1 Hz), 3.93 (s, 3H).

Methyl 3-(azidomethyl)-5-nitrobenzoate. According to method[4], triethylamine (4.32 mL, 31.0 mmol) was added to a stirring solution of methyl 3-(hydroxymethyl)-5-nitrobenzoate (4.36 g, 20.65 mmol) in CH_2Cl_2 (60 mL) at room temperature. The resulting solution was cooled to 0 °C and methanesulfonyl chloride (1.931 mL, 24.78 mmol) was added dropwise over 30 min. The solution was allowed to warm up till room temperature and stir for 30 min. The reaction mixture was successively washed with aqueous 1 N HCl (3 x 20 mL), saturated aqueous $NaHCO_3$ (3 x 20 mL), water (20 mL), brine (2 x 20 mL), dried over anhydrous $MgSO_4$, filtered and concentrated. This material was solubilised in DMF (60 mL) at room temperature and sodium azide (2.2 g, 33.8 mmol) was added in one portion under stirring. The mixture was heated at 50 °C for 2 h and DMF was removed as much as possible under vacuum. The remaining material was taken in water (150 mL) and extracted with EtOAc (3 x 50 mL). The combined organic layers were washed with water (50 mL), brine (2 x 50 mL), dried over anhydrous Na_2SO_4, filtered and concentrated. The crude material was adsorbed on celite and purified by automated chromatography (EtOAc in hexanes) to give methyl 3-(azidomethyl)-5-nitrobenzoate as a yellow oil (4.44 g, 18.78 mmol, 91%). Spectral data were consistent

with a previous report[5]. 1H NMR (CDCl3, 400 MHz) δ (ppm) 8.83 (t, 1H, J = 1.8 Hz), 8.39 (t, 1H, J = 2.0 Hz), 8.33 (t, 1H, J = 1.6 Hz), 4.58 (s, 2H), 4.00 (s, 3H).

Methyl 3-amino-5-(azidomethyl)benzoate. According to method[6], sodium hydrosulfite (5.9 g, 33.9 mmol) was added to a stirring solution of methyl 3-(azidomethyl)-5-nitrobenzoate (2.0 g, 8.47 mmol) in ethanol (80 mL) at room temperature. The suspension was heated at reflux for 24 h. More sodium hydrosulfite (5.9 g, 33.9 mmol) was added every two hours from the start till a total of 29.5 g were added. The suspension was filtered on celite with EtOAc and the filtrate was concentrated. The crude material was adsorbed on celite and purified by automated chromatography (EtOAc gradient in hexanes) to give methyl 3-amino-5-(azidomethyl)benzoate as an orange oil (954 mg, 4.63 mmol, 55%). 1H NMR (DMSO-d6, 400 MHz) δ (ppm) 7.16 (t, 1H, J = 2.0 Hz), 7.07 (t, 1H, J = 1.6 Hz), 6.77 (t, 1H, J = 1.8 Hz), 5.52 (s, 2H), 4.36 (s, 2H), 3.81 (s, 3H).

Methyl 3-(azidomethyl)-5-(furan-2-carboxamido)benzoate. According to method[7], trimethyl phosphite (0.82 mL, 6.94 mmol) was dissolved in CH_2Cl_2 (9.0mL). The solution was stirred and cooled to 0 °C then iodine (1.7 g, 6.70 mmol) was added in one portion. Once dissolved, 2-furoic acid (824 mg, 7.35 mmol) and then triethylamine (2.0 mL, 14.35 mmol) were added. After 15 min of additional stirring, 3-amino-5-(azidomethyl)benzoate (954 mg, 4.63 mmol) was added dropwise as a solution in CH_2Cl_2 (6.0 mL). After 15 min of additional stirring, the mixture was allowed to warm up till room temperature and stir overnight. CH_2Cl_2 (50 mL) was added; the organic layer was washed with saturated aqueous $NaHCO_3$ (3 x 20 mL), 1 N aqueous HCl (3 x 20 mL), water (20 mL), brine (2 x 20mL), dried over anhydrous $MgSO_4$, filtered and adsorbed on celite. Purification by automated chromatography (EtOAc gradient in hexanes) gave methyl 3-(azidomethyl)-5-(furan-2-carboxamido)benzoate as an orange oil (985 mg, 3.28 mmol, 71%). 1H NMR (DMSO-d6, 400 MHz) δ (ppm) 10.47 (s, 1H), 8.39 (m, 1H), 8.09 (s, 1H), 7.97 (d, 1H, J = 1.6 Hz), 7.69 (s, 1H), 7.39 (d, 1H, J = 3.6 Hz), 6.72 (dd, 1H, J = 1.6, 3.5 Hz), 4.59 (s, 2H), 3.88 (s, 3H).

Methyl 3-(aminomethyl)-5-(furan-2-carboxamido)benzoate. Pearlman's catalyst (218 mg, 0.310 mmol) was added to a stirring solution of methyl 3-(azidomethyl)-5-(furan-2-carboxamido)benzoate (865 mg, 2.88 mmol) in EtOAc (25 mL) at room temperature. Nitrogen was bubbled in the solution for 10 min; the reaction mixture was evacuated quickly and put under hydrogen for 3 h. The mixture was diluted with CH_2Cl_2 and filtered on celite. Solvents were removed under vacuum and the crude material was adsorbed on celite to be purified by automated chromatography (2% NH_4OH, MeOH gradient in CH_2Cl_2). Methyl 3-(aminomethyl)-5-(furan-2-carboxamido)benzoate was obtained as a white solid (548 mg, 2.00 mmol, 69%). 1H NMR (DMSO-d6, 300 MHz) δ (ppm) 10.34 (s, 1H), 8.27 (t, 1H, J = 1.8 Hz), 8.00 (t, 1H, J = 1.7 Hz), 7.95 (dd, 1H, J = 0.8, 1.7 Hz), 7.71 (t, 1H, J = 1.5 Hz), 7.38 (dd, 1H, J = 0.7, 3.4 Hz), 6.71 (dd, 1H, J = 1.7, 3.4 Hz), 3.86 (s, 3H), 3.79 (s, 2H), 2.37 (br s, 2H). MS (APCI+) calculated for $[C_{14}H_{14}N_2O_4 + H]^+$: 275.1, found: 275.1.

Methyl 3-((2,3-dihydrobenzofuran-2-carboxamido)methyl)-5-(furan-2-carboxamido)benzoate. 2,3-dihydrobenzofuran-2-carboxylic acid (94 mg, 0.573 mmol) and HATU (171 mg, 0.450 mmol) were dissolved in DMF (1.5 mL) at room temperature. The solution was stirred when DIPEA (320 μL, 1.832 mmol) and then methyl

3-(aminomethyl)-5-(furan-2-carboxamido)benzoate (98 mg, 0.357 mmol) as a solution in DMF (1.5 mL) were added. After 1 h of additional stirring, aqueous 5% wt citric acid (30 mL) was added and the mixture was extracted with EtOAc (3 x 10 mL). The combined organic layers were washed with water (10 mL), brine (2 x 10 mL), dried over anhydrous Na_2SO_4, filtered and concentrated. The crude material was adsorbed on celite and purified by automated chromatography (EtOAc in hexanes). After lyophilization, methyl 3-((2,3-dihydrobenzofuran-2-carboxamido)methyl)-5-(furan-2-carboxamido)benzoate was obtained as a white solid (105 mg, 0.250 mmol, 70%). 1H NMR (DMSO-d6, 300 MHz) δ (ppm) 10.39 (s, 1H), 8.81 (t, 1H, J = 6.1 Hz), 8.30 (t, 1H, J = 1.7 Hz), 7.96 – 7.94 (m, 2H), 7.59 (t, 1H, J = 1.5 Hz), 7.38 (dd, 1H, J = 0.9, 3.6 Hz), 7.24 – 7.21 (m, 1H), 7.13 – 7.10 (m, 1H), 6.89 – 6.80 (m, 2H), 6.71 (dd, 1H, J = 1.7, 3.4 Hz), 5.23 – 5.15 (m, 1H), 4.43 – 4.26 (m, 1H), 3.86 (s, 3H), 3.57 – 3.48 (m, 1H), 3.28 – 3.21 (m, 2H). MS (APCI+) Calculated for $[C_{23}H_{20}N_2O_6 + H]^+$: 421.1, found: 421.2.

Methyl 3-amino-5-((3,5-dimethylbenzamido)methyl)benzoate. Pearlman's catalyst (33.8 mg, 0.241 mmol) was added in one portion to a stirring solution of methyl 3-(azidomethyl)-5-nitrobenzoate (1.0 g, 4.23 mmol) in ethyl acetate (40 mL). Nitrogen was bubbled in the stirring mixture for 10 min and the atmosphere was switched for hydrogen. After stirring overnight, the mixture was diluted with DCM and filtered on celite. The filtrate was concentrated to give methyl 3-amino-5-(aminomethyl)benzoate as a pale yellow oil (839 mg, crude material). N-(3-dimethylaminopropyl)-N′-ethylcarbodiimide hydrochloride (728 mg, 3.80 mmol) and then 1-hydroxybenzotriazole hydrate (590 mg, 3.85 mmol) were added to a solution of 3,5-dimethylbenzoic acid (458 mg, 3.05 mmol) in DMF (11.5mL). The mixture was cooled to 0 °C when 3-amino-5-(aminomethyl)benzoate (533 mg, crude material) was added dropwise as a solution in DMF (3.5 mL). Then, DIPEA (2.0 mL, 11.45 mmol) was added dropwise, the cooling bath was removed and the mixture was allowed to warm up till room temperature and stir overnight. Water (90 mL) was added and the aqueous layer was extracted with EtOAc (3 x 30 mL). The combined organic layers were washed with water (30 mL), brine (2 x 30 mL), dried over anhydrous Na_2SO_4, filtered and concentrated. The crude material was adsorbed on celite and purified by automated chromatography (MeOH gradient in CH_2Cl_2) to give methyl 3-amino-5-((3,5-dimethylbenzamido)methyl)benzoate as a white solid (368 mg, 1,178 mmol, 44%). 1H NMR (DMSO-d6, 400 MHz) δ (ppm) 8.90 (t, 1H, J = 6.1 Hz), 7.50 (s, 2H), 7.16 (s, 1H), 7.06 (d, 2H, J = 2.0 Hz), 6.74 (t, 1H, J = 1.8 Hz), 5.38 (s, 2H), 4.35 (d, 2H, J = 5.8 Hz), 3.78 (s, 3H), 2.32 (s, 6H). MS (APCI+) Calculated for $[C_{18}H_{20}N_2O_3 + H]^+$: 313.1, found: 313.2.

Methyl 3-((3,5-dimethylbenzamido)methyl)-5-(furan-2-carboxamido)benzoate. Methyl 3-amino-5-((3,5-dimethylbenzamido)methyl)benzoate (19.5 mg, 0.062 mmol) was dissolved in CH_2Cl_2 (1.0 mL) at room temperature. The solution was stirred when DMAP (1.5 mg, 0.012 mmol) was added in one portion. Then, furan-2-carbonyl chloride (9 µL, 0.091 mmol) and DIPEA (60 µL, 0.344 mmol) were each added dropwise in that order. After 1 h of additional stirring, the mixture was concentrated. The crude material was dissolved in DMF (5% water) to be purified by preparative HPLC (MeOH gradient in water with 0.1% of formic acid). After lyophilisation, methyl 3-((3,5-dimethylbenzamido)methyl)-5-(furan-2-carboxamido)benzoate was obtained as a white solid (12.2 mg, 0.030 mmol, 48%). 1H NMR (DMSO-d6, 300 MHz) δ (ppm) 10.40 (s, 1H), 9.03 (t, 1H, J = 6.1 Hz), 8.33 (t, 1H, J

= 1.8 Hz), 7.99 (t, 1H, J = 1.8 Hz), 7.94 (dd, 1H, J = 0.7, 1.7 Hz), 7.66 (t, 1H, J = 1.5 Hz), 7.52 (s, 2H), 7.37 (dd, 1H, J = 0.8, 3.4 Hz), 7.17 (s, 1H), 6.70 (dd, 1H, J = 1.7, 3.4 Hz), 4.50 (d, 2H, J = 5.9 Hz), 3.86 (s, 3H), 2.32 (s, 6H). MS (APCI+) Calculated for $[C_{23}H_{22}N_2O_5 + H]^+$: 407.2, found: 407.2.

SUPPLEMENTARY REFERENCES

1 Tubeleviciute-Aydin, A. *et al.* Rare human Caspase-6-R65W and Caspase-6-G66R variants identify a novel regulatory region of Caspase-6 activity. *Sci. Rep.* **8,** 4428 (2018).

2 Mancuso, L. *et al.* Bioreduction of aryl azides during mutasynthesis of new ansamitocins. *Org. Lett.* **15,** 4442-4445 (2013).

3 Fyfe, M. C. T. *et al.* Urea derivatives useful as kinase inhibitors. WO2015092423A1 (2015).

4 Aquila, B., Lyne, P. & Pontz, T. Quinazolinone derivatives having b-raf inhibitory activity. US20090170849A1 (2009).

5 Watzke, A. *et al.* A generic building block for C- and N-terminal protein-labeling and protein-immobilization. *Bioorg. Med. Chem.* **14,** 6288-6306 (2006).

6 Yang, D., Fokas, D., Li, J., Yu, L. & Baldino, C. M. A Versatile Method for the Synthesis of Benzimidazoles from o-Nitroanilines and Aldehydes in One Step via a Reductive Cyclization. *Synthesis* **2005,** 47-56 (2005).

7 Luo, Q.-L. *et al.* An Efficient Protocol for the Amidation of Carboxylic Acids Promoted by Trimethyl Phosphite and Iodine. *Eur. J. Org. Chem.* **2011,** 6916-6922 (2011).
